# Mechanism-to-Deployment Engineering of NO_2_ Gas Sensors: Materials, Interfaces, Transducers, and Environmental Validation

**DOI:** 10.3390/ma19183874

**Published:** 2026-09-11

**Authors:** Daewoong Jung

**Affiliations:** 1 Department of Nanomechatronics Engineering, College of Nanoscience & Nanotechnology, Pusan National University, 2 Busandaehak-ro, Busan 46241, Republic of Korea; dwjung@pusan.ac.kr; 2 School of Transdisciplinary Engineering, College of Engineering, Pusan National University, 2 Busandaehak-ro, Busan 46241, Republic of Korea

**Keywords:** NO_2_, gas sensor, chemiresistive sensing, metal oxide semiconductor, heterojunction, interface engineering, sensor integration, environmental validation

## Abstract

**Highlights:**

**What are the main findings?**

• NO_2_ sensing reflects coupled surface chemistry, interfaces, and transducer behavior.
• Heterojunctions, defects, catalysts, and active regeneration reshape sensor response.
• Humidity, flow, recovery, aging, and device variability govern practical reliability.

**What are the implications of the main findings?**

• Mechanism-to-deployment evaluation is more useful than ranking peak response alone.
• Validation should define humidity, flow, power, recovery, and calibration envelopes.
• Standardized reporting and field calibration are essential for reliable NO_2_ monitoring.

**Abstract:**

NO_2_ sensing is shifting from the optimization of individual receptor materials toward integrated systems in which surface chemistry, interfacial charge transfer, transducer architecture, operating environment, and regeneration jointly determine performance. This review argues that deployment readiness is set not by peak response but by the coupled performance of five layers: (i) receptor and interface chemistry, (ii) transducer and device architecture, (iii) gas delivery and environmental conditions, (iv) regeneration and aging, and (v) calibration, uncertainty, and system integration. Within this mechanism-to-deployment framework, we examine how oxygen adsorption, depletion and accumulation layers, heterojunction and Schottky barriers, defects, catalytic sensitization, and percolation govern the electrical signal across metal oxides, carbon materials, transition-metal dichalcogenides, MXenes, porous and MOF-derived architectures, and organic semiconductors and how MEMS microheaters, FET/TFT/MOSFET transducers, flexible platforms, optical and electrical regeneration, and AI-assisted arrays read it out. Despite this progress, translation remains limited by environmental interference, incomplete recovery, transport-dependent response, aging, and device-to-device variability so that record responses seldom survive realistic operation. Reliable NO_2_ monitoring therefore requires application-specific validation of the complete measurement cycle—exposure, readout, recovery, environmental perturbation, calibration, and long-term operation—rather than isolated sensitivity metrics.

## 1. Introduction

NO_2_ is a strongly oxidizing trace gas produced predominantly by combustion-related activities, including transportation, power generation, industrial processing, and accidental or occupational releases. Beyond its established role in photochemical smog and acid deposition, NO_2_ is increasingly treated as a spatially heterogeneous exposure variable whose concentration can change sharply across road corridors, industrial zones, indoor microenvironments, and personal activity patterns. Recent reviews also broaden the motivation for monitoring beyond human respiratory exposure by highlighting consequences for vegetation physiology, phenology, and ecosystem composition. These combined health, environmental, and ecosystem drivers favor sensing approaches that can complement fixed monitoring stations with denser, more distributed measurements [1,2].

The available sensing technologies span optical and spectroscopic analyzers, electrochemical devices, photoacoustic platforms, field-effect devices, and chemiresistive semiconductors, but they occupy different positions in the trade space of selectivity, calibration traceability, size, power, cost, and temporal resolution. Optical and photoacoustic approaches can provide strong molecular specificity and, in the case of dual-resonant photoacoustic detection, simultaneous NO/NO_2_ discrimination, but they require controlled optical excitation, resonator or path-length design, flow management, and integration or averaging time [1,3]. For distributed low-cost nodes, semiconducting chemiresistors remain especially attractive because they are structurally simple and compatible with microfabrication; however, conventional metal-oxide platforms frequently rely on thermal activation and therefore face persistent power, packaging, and integration penalties [4,5,6].

Recent work increasingly shifts NO_2_ sensor design from isolated receptor optimization toward coupled interface, transducer, and system engineering. Heterojunctions and mixed-dimensional structures use band alignment, built-in fields, defect control, catalytic sites, and photocarrier separation to amplify charge transfer, while MEMS, FET/MOSFET, flexible, and wireless platforms use thermal, electrical, or algorithmic control to shape the readout and recovery process [7,8,9,10,11,12,13,14,15]. The common design principle is that practical performance is governed by where charge is generated, transferred, depleted, regenerated, and finally measured, rather than by surface area or peak response alone.

Despite these advances, translation from a laboratory response curve to a deployable NO_2_ sensor remains limited by coupled environmental and system effects. High activation energy can penalize battery operation; water and interferents perturb adsorption and charge transport; strong NO_2_ binding can create incomplete recovery and drift; and measured magnitude and kinetics depend on flow, chamber exchange, sensor position, and exposure history [16]. Trace-concentration claims are also difficult to compare when the lowest generated concentration, signal quality, and exposure duration are not reported consistently. Low-temperature operation does not remove aging: repeated photoactivation of Ag-ZnO over 60 days altered vacancy density, Ag oxidation state, dark conductivity, and photoconductivity, directly degrading NO_2_ response [17]. These limitations make peak response alone an insufficient descriptor of deployment readiness.

The existing review literature provides important but partly segmented views of this field. Broad surveys organize NO_2_ sensors by functional material and recent performance advances [1]; other reviews concentrate on rGO-containing hybrid sensing mechanisms [18], low-temperature ZnO platforms [6], ZnO nanostructures [19], or metal-oxide sensors for industrial exhaust environments [5]. Such perspectives are valuable for material selection and mechanistic orientation, but they do not consistently connect receptor chemistry with transducer architecture, gas-delivery conditions, recovery strategy, long-term aging, signal quality, and application-specific validation. As the field moves toward flexible, low-power, photoactivated, transistor-based, AI-assisted, and wirelessly connected systems, this separation between materials science and deployment engineering becomes a growing limitation of conventional review structures.

This review is therefore positioned around a mechanism-to-deployment framework rather than a material-performance ranking. NO_2_ sensing is evaluated across five coupled layers: receptor chemistry, interface control, transducer design, environmental and gas-delivery conditions, and deployment metrics. The analysis preserves each paper’s original response definition, distinguishes experimentally tested concentrations from calculated or extrapolated limits, and treats humidity, flow, recovery completeness, aging, device variability, and signal quality as measurement variables rather than secondary details. The goal is not to identify a universally best material but to determine which combinations of mechanism, architecture, and validation are credible for a stated application.

Accordingly, Section 2 defines the literature scope and review methodology; Section 3 develops the fundamental sequence of adsorption, charge transfer, barrier modulation, percolative transport, and regeneration; Section 4 compares functional-material families and interface-engineering strategies; Section 5 examines device architectures, transduction, active regeneration, and system integration; Section 6 evaluates environmental effects, selectivity, recovery, transport conditions, reliability, and calibration transfer; Section 7 establishes a framework for performance benchmarking, reporting, and application-specific validation; Section 8 identifies research gaps and translation priorities spanning mechanism verification, life-cycle energy, field robustness, and manufacturability; and Section 9 concludes with deployment-oriented design priorities.

## 2. Scope, Literature Coverage, and Review Methodology

This review focuses on NO_2_ sensing strategies relevant to low-temperature, room-temperature, portable, flexible, or otherwise deployment-oriented operation. The scope includes chemiresistive metal oxides and heterostructures; carbon/oxide hybrids; TMDs, MXenes, g-C_3_N_4_, MOF-derived and porous architectures; conducting and organic semiconductors; MEMS and transistor-based devices; photoactivated and pulse-regenerated sensors; and selected photoacoustic or system-integrated platforms. The emphasis is on experimentally demonstrated behavior and on how material design, transducer architecture, operating protocol, and environment jointly shape practical performance.

Key comparison descriptors are recorded when reported by the source: material/composition, transducer architecture, operating and activation conditions, original response definition, experimentally tested concentration range, response/recovery kinetics, humidity and interferent conditions, regeneration method, gas-delivery/flow conditions, long-term stability, and device- or system-level validation. For flexible systems, mechanical test conditions are considered together with sensing performance; for heater-, light-, or bias-assisted devices, activation and recovery are treated as part of the measurement cycle.

Cross-study comparison is deliberately conservative because response equations, gas delivery, humidity, exposure duration, device geometry, and regeneration protocols differ widely. Reported responses are therefore preserved in their original form unless the baseline, denominator, and direction of change are explicit. Trace performance is separated into (i) the lowest experimentally tested concentration, (ii) a calculated or extrapolated limit of detection when that is what the source reports, and (iii) a noise-equivalent concentration only when the original study provides sufficient noise/SNR information; missing values are not inferred. Results obtained under different flow or chamber conditions are not treated as directly interchangeable.

## 3. Fundamental Sensing Mechanisms and Signal Formation

The sensing signal in a NO_2_ device should be interpreted as a sequence rather than as a single surface reaction. Gas transport establishes the analyte flux at the receptor; adsorption and surface chemistry redistribute charge; depletion, accumulation, interfacial barriers, contacts, or percolating networks convert that charge redistribution into a conductivity change; and desorption or active regeneration restores the baseline. Reviews of metal oxides, oxide heterojunctions, rGO hybrids, van der Waals materials, and CNT interfaces all converge on this multiscale picture, although the dominant step differs with material and transducer architecture [1,4,5,18,20,21,22,23,24].

### 3.1. Surface Adsorption, Oxygen Ionosorption, and Charge Transfer

For conventional chemiresistive metal oxides, the air baseline is established by oxygen adsorption and charge exchange with the semiconductor. Adsorbed O_2_ withdraws electrons from n-type materials such as ZnO, SnO_2_, In_2_O_3_, and WO_3_, creating a near-surface electron-depletion region and increasing grain-boundary barriers. In p-type oxides, the same electron withdrawal increases the near-surface hole population and can form a hole-accumulation layer. The measured baseline resistance therefore reflects the coupled bulk/surface state of the film rather than the intrinsic conductivity of the oxide alone [1,4,5,19,20].

A commonly used surface-ionization model assigns molecular O_2_^−^ to lower-temperature conditions, O^−^ to intermediate temperatures, and more strongly ionized oxygen states to higher temperatures. These temperature windows are useful as a conceptual framework, but the actual surface population depends on crystallographic facet, defect chemistry, humidity, carrier density, and measurement atmosphere. Recent SnO_2_ studies in particular show that surface vacancy complexes, faceting, and operating temperature must be considered together when interpreting NO_2_ response [6,20,25,26].

O_2_(g) → O_2_(ads)

O_2_(ads) + e^−^ → O_2_^−^(ads)   (commonly associated with lower-temperature adsorption)

O_2_^−^(ads) + e^−^ → 2O^−^(ads)  (commonly associated with intermediate temperatures)

O^−^(ads) + e^−^ → O^2−^(ads)  (higher-temperature surface state in the conventional model)

NO_2_ is a strong electron acceptor and can interact with the receptor by direct electron capture and through reactions involving preadsorbed oxygen or surface hydroxyl species. In an n-type current path, additional electron withdrawal expands depletion and commonly increases resistance; in a p-type current path, it increases hole density and commonly decreases resistance. The same chemical direction of charge transfer can therefore produce opposite electrical polarities depending on the dominant carrier and on whether current is controlled by a grain, a junction, a contact, or a conducting network. This carrier-sign principle extends beyond oxides to CNT, graphene, conducting-polymer, and layered-semiconductor sensors [18,20,24,27,28,29,30,31,32].

Microstructure determines how efficiently this surface charge perturbation reaches the electrical readout. When grain diameter, nanowire radius, or intergrain neck width becomes comparable with the depletion scale, a larger fraction of the conduction cross-section is controlled by adsorbate-induced charge. Porosity increases the accessible internal area and reduces the diffusion distance, whereas dense agglomeration can shield active surfaces or produce poorly controlled current paths. Thus, nanoscale size, neck geometry, pore connectivity, facet exposure, and contact topology should be interpreted as transduction variables as well as morphological descriptors [19,25,33,34,35,36,37,38,39].

Baseline restoration requires the adsorbed nitrogen-containing species and associated surface charge to relax toward the original air state. At low temperatures, adsorption can remain sufficiently strong to generate a large signal, while desorption is kinetically slow, leading to hysteresis and baseline drift. This is why recovery should be treated as part of the sensing mechanism itself and why thermal, optical, or electrical reset strategies are increasingly integrated into low-temperature sensors [20,30,32,40,41].

**Figure 1:**
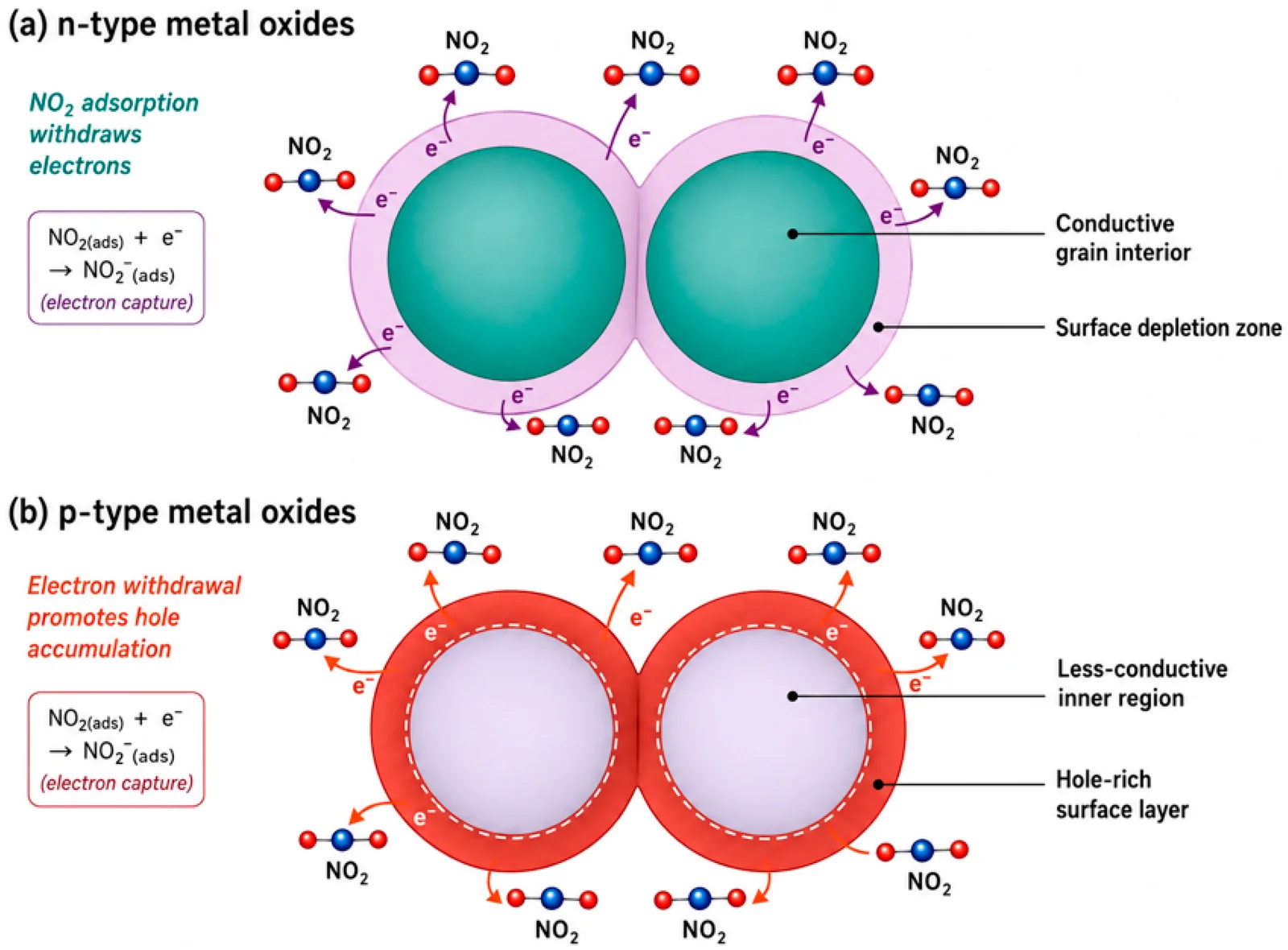
Schematic illustration of NO_2_ sensing in n-type and p-type metal oxides. (**a**) NO_2_ adsorption on n-type oxides withdraws electrons and expands the surface depletion region. (**b**) NO_2_ adsorption on p-type oxides promotes hole accumulation at the surface. This schematic was prepared with the assistance of ChatGPT (OpenAI, GPT-5.6)) and was subsequently reviewed, edited, and verified by the author.

Figure 1 compares the common electron-acceptor pathway in n-type and p-type metal oxides. In n-type oxides, NO_2_-induced electron withdrawal expands the surface depletion zone and increases resistance; in p-type oxides, the same electron withdrawal increases the hole-rich surface layer and lowers resistance. The opposite electrical polarity therefore arises from the dominant carrier type even though the direction of interfacial charge transfer is the same.

### 3.2. Heterojunction and Schottky Barrier Modulation with Percolative Transport

Junction engineering introduces an additional electrostatic transduction step. When two semiconductors or a metal and a semiconductor with different work functions or Fermi levels are brought into contact, carriers redistribute until electrochemical equilibrium is reached. The resulting band bending forms depletion/accumulation regions, a built-in p-n or n-n barrier, or a Schottky barrier. NO_2_-induced charge transfer then changes the height or width of this pre-existing barrier, allowing a modest surface charge change to generate a much larger current modulation when the interface lies on the dominant conduction path [21,22,23,42,43,44,45,46].

The magnitude of junction amplification depends less on the nominal presence of a second phase than on current-path topology. A low loading may create too few electronically active interfaces, whereas excessive conductive carbon, MXene, or metallic loading can bypass the junction, short-circuit the barrier-controlled pathway, or shield adsorption sites. This explains the recurring optimum-composition behavior reported for oxide/oxide, oxide/carbon, oxide/MXene, and mixed-dimensional hybrids [27,34,35,46,47,48,49,50,51,52,53,54,55,56,57,58].

**Figure 2:**
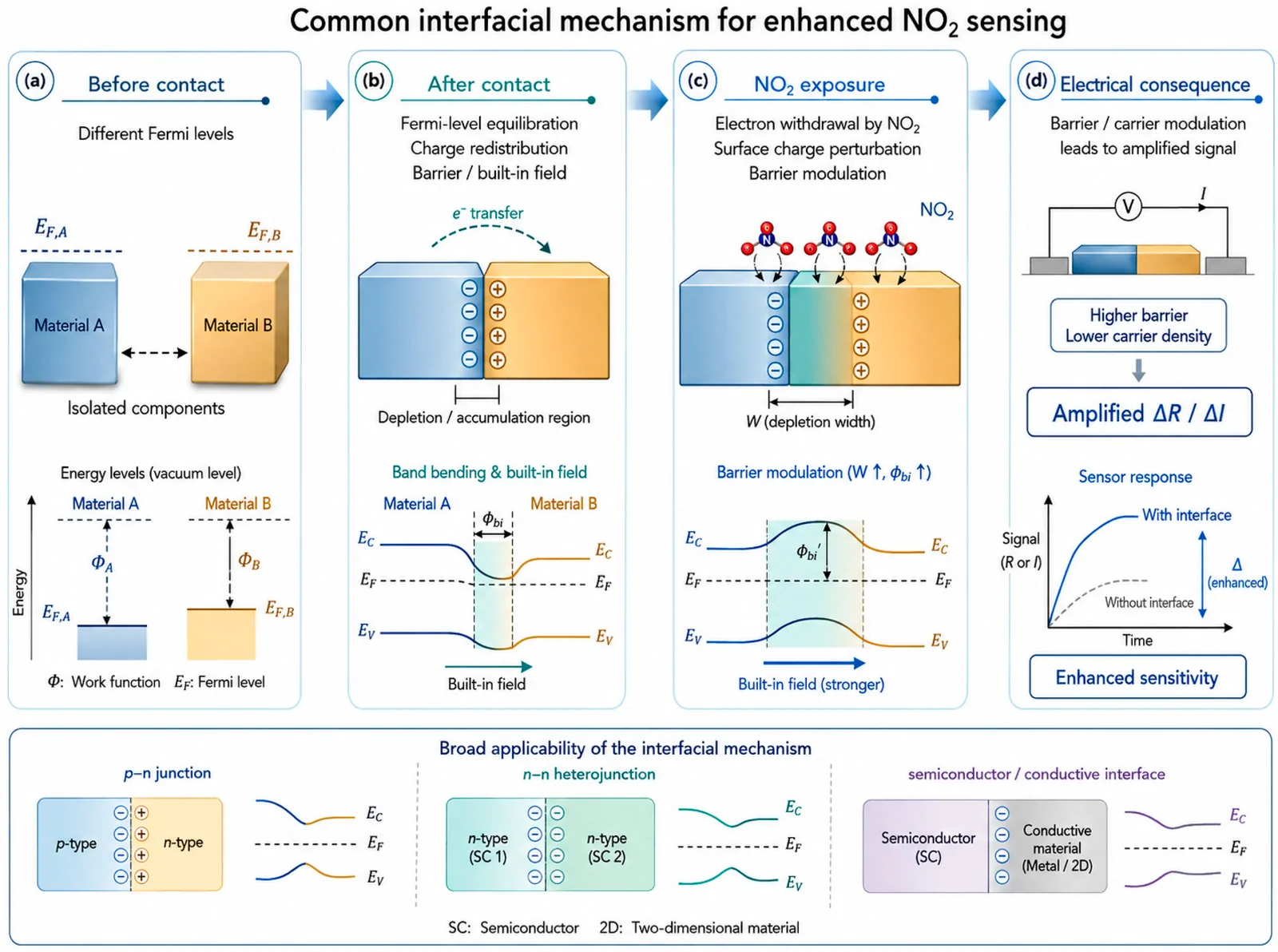
Conceptual summary of the common interfacial mechanism for enhanced NO_2_ sensing. (**a**) Fermi-level/work-function mismatch before contact. (**b**) Charge redistribution, band bending, and built-in field formation after contact. (**c**) NO_2_-induced modulation of depletion/accumulation and the interfacial barrier. (**d**) Amplified electrical response. The framework applies to p–n, n–n, and semiconductor/conductive interfaces, including CuO/ZnO [42], WS_2_/SnO_2_ [43], In_2_O_3_/In_2_S_3_ [45], and PTCDI/Ti_3_C_2_T_x_ MXene [46]. This schematic was prepared with the assistance of ChatGPT (OpenAI) and was subsequently reviewed, edited, and verified by the author.

Figure 2 summarizes the common interfacial mechanism underlying enhanced NO_2_ sensing in heterostructured materials. Before contact, dissimilar materials possess different Fermi levels and work functions. Upon contact, Fermi-level equilibration drives charge redistribution, resulting in band bending, interfacial depletion or accumulation, and the formation of a built-in potential. Subsequent NO_2_ adsorption withdraws electrons and perturbs the interfacial charge distribution, thereby modulating the depletion width and/or barrier height. This interfacial modulation provides an additional contribution to the electrical response beyond surface adsorption alone. A representative p-CuO/n-ZnO system illustrates this framework: NO_2_ exposure alters both the hole population in p-CuO and the junction potential so that the measured resistance change reflects coupled surface adsorption and interface barrier effects [42]. Consequently, heterostructure composition, junction type, and the position of the interface within the current-conduction pathway become key design variables for maximizing NO_2_ sensitivity. The same general framework can be extended to p–n junctions, n–n heterojunctions, and semiconductor/conductive-material interfaces, although the direction and magnitude of barrier modulation depend on the specific electronic structures of the constituent materials.

Across the wider literature, the same principle appears in p-n systems such as CuO/CeO_2_, NiO-SnO_2_, CuO/ZnO, and CuO/TiO_2_; in n-n or oxide–sulfide systems such as SnO_x_/g-C_3_N_4_, In_2_S_3_/In_2_O_3_, and SnO_2_/SnS_2_; and in mixed-dimensional structures containing MoS_2_, WS_2_, SnSe_2_, graphene, or MXenes [7,8,9,10,27,42,43,44,45,46,49,58,59,60,61,62,63,64,65,66,67]. These studies should be interpreted through the same question: does NO_2_ modulate an interface that actually controls a significant fraction of the device current?

### 3.3. Defect Engineering, Surface Vacancies, and Catalytic Sensitization

Defects affect NO_2_ sensing by changing both chemical reactivity and semiconductor electrostatics. Oxygen vacancies, under-coordinated atoms, dopants, and high-index facets can alter the density and binding strength of adsorption sites while also changing donor/acceptor density, local work function, and depletion width. Because these effects are coupled, a larger vacancy signal in spectroscopy does not automatically imply a better sensor; the useful defect population is the one that increases reversible surface interaction without producing excessive baseline conduction, instability, or irreversible adsorption [25,26,37,39,68,69,70,71,72,73,74,75].

Catalytic functionalization adds a related but distinct route. Noble metals can promote chemical sensitization by activating oxygen or NO_2_ and transferring reactive species to the semiconductor surface (spillover), while work-function mismatch can create electronic sensitization through a gas-responsive Schottky barrier. Similar logic applies to catalytic filter layers positioned upstream of the receptor: instead of amplifying the receptor directly, the filter selectively transforms or suppresses interferents before they reach the sensing element [43,47,76,77,78,79,80,81,82,83].

Both defect engineering and catalytic sensitization therefore exhibit an optimum rather than a monotonic dependence on loading. Excess catalyst can block adsorption sites, create parallel metallic conduction, or bind species too strongly for rapid regeneration; excessive vacancy density can increase drift or make the baseline overly sensitive to oxygen and humidity. The most informative studies combine spectroscopy, electrical response, and controlled composition so that morphology, defect density, and electronic transport are not conflated [17,25,39,43,78,80,82,83].

Non-metallic modifiers broaden this concept further. Polyoxometalate electron acceptors, heteroatom-doped carbon, amine-terminated surfaces, and organic/inorganic hybrid interfaces can regulate trapping, carrier separation, and adsorption affinity without relying on a noble-metal catalyst. Their contribution is best evaluated by asking whether the modifier creates a reversible chemical interaction, a useful electrostatic barrier, or a more favorable current pathway rather than simply increasing nominal surface functionality [10,27,28,46,84,85].

**Figure 3:**
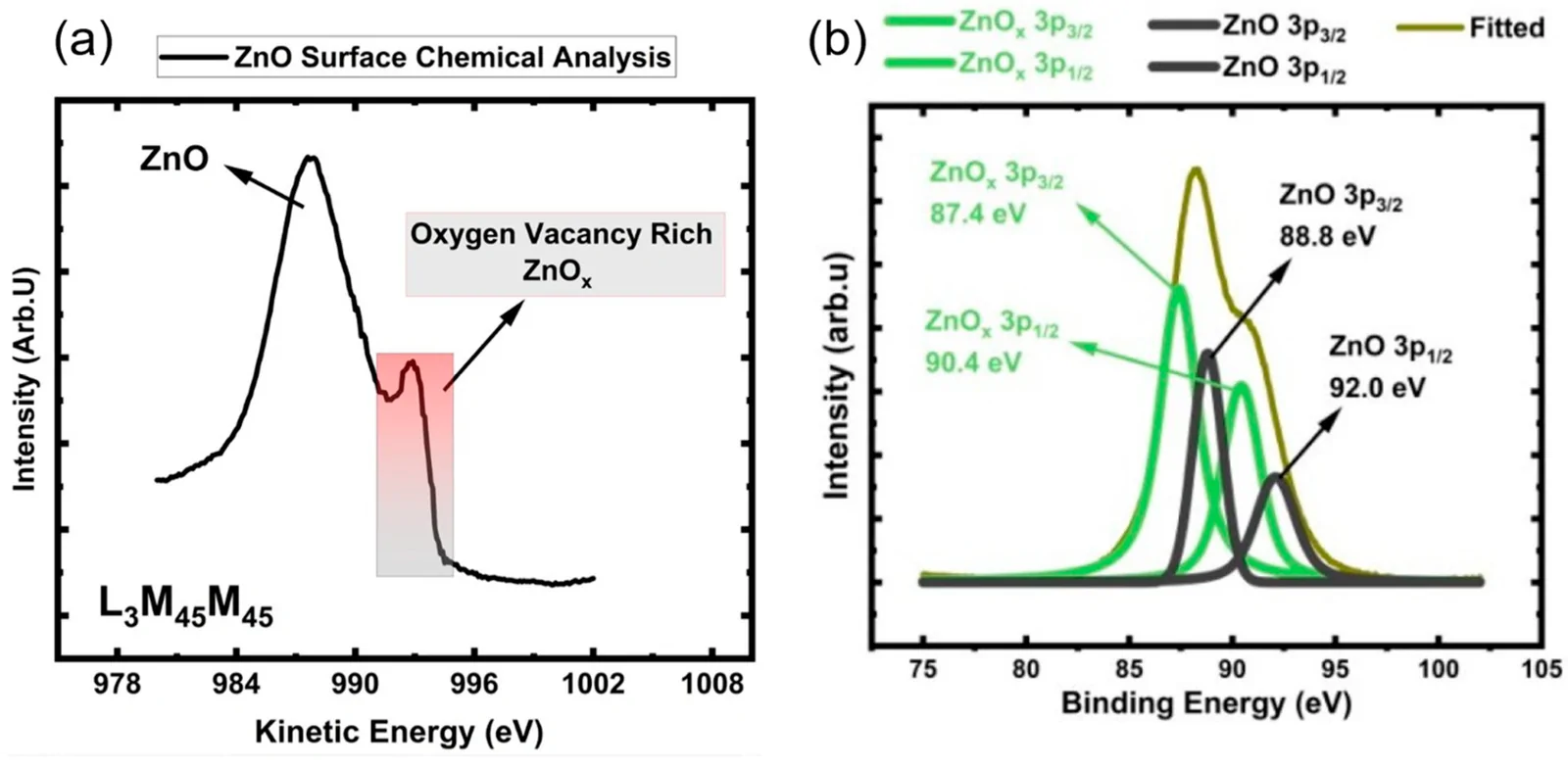
(**a**) ZnO L_3_M_45_M_45_ position with 1147 eV excitation X-ray photons and (**b**) deconvoluted Zn 3p peak, showing non-stoichiometric ZnO_x_ associated with oxygen vacancies. All panels adapted with permission from Ref. [39].

Figure 3 connects defect chemistry with accessible morphology in engineered-porosity ZnO. The Auger and Zn 3p spectra identify a non-stoichiometric, vacancy-rich surface contribution, while the open, tortuous pore network exposes a larger fraction of the ZnO volume to the gas phase. The sensing enhancement is therefore a coupled transport-and-chemistry effect: porosity governs how efficiently NO_2_ reaches the active solid, and the vacancy-rich surface governs how strongly that accessible area exchanges charge with adsorbates [39].

The Au-decorated WS_2_/SnO_2_ system [43] provides a representative example of how interface and catalyst states jointly shape the electrical landscape underlying NO_2_ sensing. UPS measurements establish a work-function difference between WS_2_ and SnO_2_, supporting n–n heterojunction formation and band bending, while Au decoration introduces local metal–semiconductor barriers and catalytic surface sites. NO_2_-induced electron withdrawal can therefore modulate both surface carrier density and interfacial barrier height. This study complements the generalized framework in Figure 2 and illustrates why catalyst loading and interface placement should be considered together rather than as independent adsorption-site effects [43].

### 3.4. Adsorption–Desorption Kinetics, Activation, and Signal Regeneration

Sensor transients arise from several coupled time constants: chamber exchange and boundary-layer transport, diffusion through the sensing layer, adsorption and surface reaction, charge equilibration, carrier transport through grains or interfaces, and finally desorption during purge. The measured response or recovery time can therefore be limited by the test cell rather than by intrinsic surface kinetics. Direct flow rate studies and controlled SnO_2_ experiments reinforce the need to interpret kinetic values together with flow, chamber geometry, exposure duration, film porosity, and operating temperature [16,20,25,38,86,87].

Temperature changes several of these steps at once. Moderate heating can increase reaction rates and accelerate desorption, but it also changes the surface coverage of oxygen, NO_2_-derived species, and water; the resulting response commonly shows an optimum rather than a monotonic trend. Temperature-programmed MEMS arrays exploit this dependence deliberately: the same SnO_2_ receptor can produce different temporal fingerprints at different heater states, converting operating temperature from a nuisance variable into an additional selectivity dimension [5,25,26,33,72,87].

Optical and electrical activation provide alternative ways to control adsorption and regeneration. Continuous illumination changes carrier population and surface coverage during the measurement, whereas a recovery-only light, heat, or gate-bias pulse leaves the analytical exposure largely unperturbed and supplies energy only when desorption is needed. Examples include UV-conditioned graphene, visible-light-reset In_2_O_3_, photoactivated oxide/2D hybrids, pulsed-bias In_2_O_3_ TFTs, localized or memristive heaters, and FePcF_16_–rGO operated with UV-assisted recovery [13,17,30,32,40,41,81,88,89,90,91].

Taken together, the reviewed literature supports a mechanism-first interpretation in which adsorption chemistry, defect/facet state, junction or contact electrostatics, current-path topology, and regeneration kinetics are treated as coupled variables. A large response may originate from more adsorbed NO_2_, stronger depletion, a more sensitive barrier, a percolation threshold, or higher transducer gain; several may coexist in one device. Mechanistic claims are strongest when the electrical signal is linked to independent surface, structural, or work-function measurements under conditions close to those used for sensing [1,18,20,21,22,24]. The fundamental sensing mechanisms discussed in Section 3 are summarized in Table 1, linking each mechanism to representative systems and its principal design implications and limitations.

**Table 1:** Summary of fundamental NO_2_ sensing mechanisms and their practical implications.

Mechanism/Process	Key Role in NO_2_ Sensing	Representative Systems	Design Implication/Limitation	References
Surface oxygen and direct charge transfer	Adsorbed oxygen establishes the baseline surface charge; NO_2_ then acts mainly as an electron acceptor, increasing depletion in n-type paths and hole accumulation in p-type paths.	SnO_2_, ZnO, In_2_O_3_, WO_3_; CuO/NiO; CNT, graphene, PANI/BP, InSe-graphene	Response polarity follows the dominant carrier and current path. Oxygen coverage, surface hydroxylation, grain/contact geometry, and reversibility determine how strongly charge transfer is expressed.	[1,4,5,6,18,19,20,24,25,26,27,28,29,30,31,32]
p–n/n–n/Schottky barrier modulation	Fermi-level equilibration creates depletion/accumulation regions and built-in barriers. NO_2_-induced charge transfer changes barrier height or width and can amplify conductance changes.	CuO-ZnO, NiO-SnO_2_, WS_2_/SnO_2_, WS_2_/WO_3_, In_2_O_3_/In_2_S_3_, PTCDI/MXene	Barrier amplification is effective only when the interface lies on the dominant current path; second-phase loading and contact geometry therefore show an optimum.	[7,8,9,10,21,22,23,42,43,44,45,46,49,58,59,60,61,62,64,65,66]
Percolation and conductive-network control	Carbon or MXene phases reduce baseline resistance and route current through gas-responsive interfaces, contacts, or tunneling junctions.	CNT/SnO_2_, rGO/oxide hybrids, N-rGO/NiO, PTCDI/MXene, InSe-graphene	Insufficient loading gives poor connectivity, whereas excessive conductive phase can shunt barrier modulation or shield adsorption sites.	[24,27,31,34,35,46,47,48,50,51,52,53,54,55,56,57,58]
Defects, vacancies, and faceting	Vacancies, dopants, under-coordinated atoms, and high-index facets modify both adsorption-site populations and semiconductor carrier density.	Y/Ag/Co-WO_3_, porous ZnO, Dy-SnO_2_, defect-tailored SnO_2_, TiO_2_-QD/In_2_O_3_	The useful defect population must improve reversible surface chemistry without destabilizing baseline conduction or excessively slowing desorption.	[17,25,26,37,39,68,69,70,71,72,73,74,75,76]
Catalytic sensitization, spillover, and chemical filtering	Noble metals can catalyze adsorption/reaction and create Schottky barriers; upstream catalytic filters can suppress interferents before they reach the receptor.	Pd-SnO_2_/rGO, Au-WS_2_/SnO_2_, Ag-WO_3_, Au/Ga_2_O_3_, ZnO-filter/Pd-SnO_2_	Catalyst size, coverage, and filter placement must balance chemical activation/selectivity against blocked area, parallel conduction, crosstalk, and slow desorption.	[43,47,76,77,78,79,80,81,82,83]
Electron acceptors, traps, and organic/inorganic modifiers	Electron-accepting or functional surface species regulate carrier trapping, interfacial transfer, adsorption affinity, and recombination.	PW_12_/SnS_2_, Co_3_O_4_/SiW_12_, APTES/WO_3_-x, N-rGO/NiO, PANI/BP, PTCDI/MXene	Trap density and interfacial fraction have an optimum range; functionalization should be judged by reversibility and baseline stability as well as response.	[10,27,28,46,84,85]
Adsorption–regeneration kinetics and active reset	Gas delivery, diffusion, adsorption/reaction, charge equilibration, electrical transport, and desorption form a coupled kinetic chain; thermal, optical, or electrical actuation can accelerate slow steps.	Visible-light In_2_O_3_, UV graphene, FePcF_16_–rGO, SnO_2_ MEMS arrays, In_2_O_3_ TFT, localized/memristive heaters	Response/recovery time should be interpreted with flow, exposure duration, recovery fraction, activation timing, and cumulative thermal/optical/electrical dose.	[13,16,17,30,32,40,41,81,87,88,89,90,91]

## 4. Functional Materials and Interface-Engineering Strategies

### 4.1. ZnO: Morphology, Defect Engineering, and Catalytic/Heterointerface Control

ZnO remains a model n-type NO_2_ receptor because morphology, defect density, grain-boundary barriers, and surface catalysts can be tuned over a wide range [6,19]. MOF-derived porous ZnO [36] demonstrates the advantage of connected pores and exposed active sites, although its strongest result requires 200 °C. CuO/ZnO microspheres [60] reduce the optimum temperature through p-n barrier modulation. The earlier ZnO nanowall/rGO sensor [34] established room-temperature heterojunction operation with 25/15 s response/recovery, while Ti_3_C_2_Tx@ZnO [55] added conductive MXene pathways and multiple Schottky barriers at 80 °C.

The carbon-templated rough-layer ZnO study [40] extends morphology engineering beyond powder synthesis. Dewdrop-like carbon micro/nanodroplets were deposited in situ and used as a removable template, producing a hierarchical rough film rich in grain boundaries and mesopores. The sensor reached a response of 131 to 10 ppm at 180 °C and detected 5 ppb; a short UV pulse accelerated recovery. This architecture addresses film integration, aggregation, and surface accessibility in a scalable format rather than treating surface area as a powder-only property.

Noble-metal and defect engineering alter both adsorption strength and electrical transduction. Experimental ZnO@Au core–shell nanorods reduced the optimum temperature from 300 to 150 °C and shortened the 5 ppm response/recovery times from 252/112 s to 108/57 s, with experimental detection at 0.5 ppm [78]. Long-term Ag-ZnO measurements further show that catalyst state and defect population can evolve during repeated photoactivated operation, so initial catalytic enhancement should be evaluated together with aging and recovery behavior [17].

Interface uniformity is as important as nominal composition. Conformal heterogeneous stacking of mono-to-few-layer rGO on ZnO nanowires produced 8/4 s response/recovery at 100 °C and retained detection to 0.5 ppm, outperforming randomly drop-cast and noble-metal-decorated controls [92]. ZnO/g-C_3_N_4_ microspheres reached a 1463% response to 10 ppm at 180 °C, compared with 229% for pristine ZnO, through increased surface area, active-site density, and interfacial charge transfer [93]. Flexible Pt-ZnO/rGO assemblies provide an additional example of two-dimensional catalyst/oxide/carbon interface design [94]. The 1463% value should not be numerically ranked against the rough-layer ZnO response of 131, even though both were reported at 10 ppm and 180 °C, because the response conventions and device/activation protocols are not identical. These cases are therefore used to compare interface and morphology design rather than to establish a response hierarchy.

Dopants can increase vacancy-mediated affinity but may penalize recovery. One-percent Rh-doped electrospun ZnO nanofibers produced Rg/Ra = 36.17 at 10 ppm and 150 °C and were experimentally tested at 50 ppb, but recovery remained 512 s at 10 ppm and 964 s at 50 ppb [70]. Two-percent Ce-doped ZnO nanoarrays reached Rg/Ra = 34.3 at 10 ppm and 250 °C; the enhanced response was associated with Ce-induced oxygen-vacancy modification, while high humidity still reduced the response [71]. Three-dimensional nanotube graphene/ZnO composites instead used an interconnected carbon network to reduce restacking and modulate the barrier, enabling room-temperature testing to 100 ppb [95].

Device-oriented ZnO structures reveal different manufacturing trade-offs. CdTe-functionalized ZnO deposited in porous Si reduced the optimum temperature from 150 °C for ZnO@PSi to 90 °C and produced a 19.82% response to 1 ppm with 13/54 s kinetics [66]. Porous ZnO nanosheets achieved Rg/Ra = 2.93 at 0.5 ppm and 74.68 at 10 ppm at 200 °C without a second phase, attributing enhancement to pore accessibility, ZnO/ZnO homojunctions, and defects [37]. The former emphasizes hierarchical heterointerfaces; the latter shows that well-controlled single-oxide morphology can remain competitive.

Long-term defect evolution must be separated from initial sensitivity. In Ag-ZnO, repeated photoactivated operation increased oxygen-vacancy density and oxidized Ag^0^ to Ag^+^, increasing dark conductivity while decreasing photoconductivity and NO_2_ sensitivity; visible light was more stable than UV over 60 days [17]. Thus, morphology, catalytic activation, and aging of the electronic structure should be evaluated as distinct design variables.

**Figure 4:**
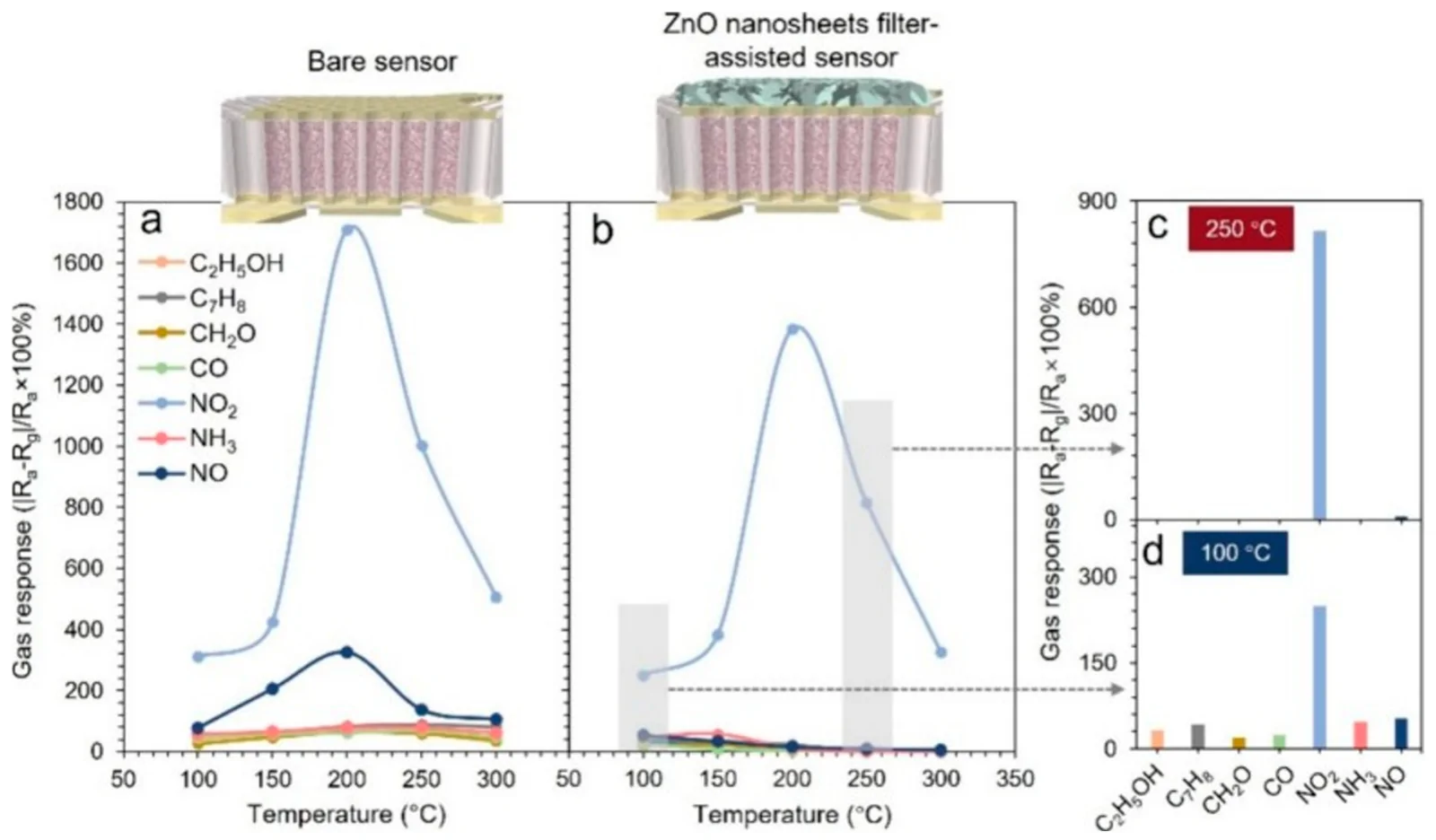
Sensing properties of bare and ZnO nanosheet filter-assisted gas sensors to 1 ppm gas species at different temperatures. (**a**) Gas response vs. working temperature of the bare gas sensor. (**b**) Gas response vs. working temperature of the ZnO nanosheet filter-assisted gas sensor. (**c**,**d**) Histograms of gas responses of ZnO nanosheet filter-assisted gas sensors at 250 and 100 °C. All panels adapted with permission from Ref. [83].

Figure 4 demonstrates that selectivity can be created by a device-level chemical pre-filter rather than by changing the receptor alone. The bare Pd/SnO_2_ nanotube sensor remains responsive to several interfering gases, whereas the reticulate ZnO nanosheet overlayer strongly suppresses responses to ethanol, formaldehyde, toluene, CO, NH_3_, and NO while preserving a large NO_2_ signal. The top–bottom electrode configuration is important because it electrically decouples the catalytic filter from the primary readout, minimizing filter-induced signal crosstalk. The same filter also attenuates humidity interference. Thus, the ZnO layer acts as a selective reaction and transport gate upstream of the SnO_2_ sensing element, illustrating a useful route to improve chemical specificity without sacrificing the stability of a conventional oxide transducer [83].

### 4.2. SnO_2_: Porosity, Heterointerfaces, Sulfide-Derived Junctions, and Conductive Hybrids

SnO_2_ is a chemically stable n-type oxide with intrinsic oxygen vacancies and a long history of scalable sol–gel, hydrothermal, sputtering, and thin-film processing. Pd-SnO_2_/rGO [47] combines porous SnO_2_, catalytic Pd, and a conductive rGO network for room-temperature detection. The sprayed rGO/SnO_2_ device [96] sacrifices speed and low-concentration performance in exchange for flexibility and a broad 20–100 ppm range. Hydrothermal SnO_2_ [33] shows that synthesis-controlled mesoporosity can lower the operating temperature to 100 °C, whereas a sol–gel/spin-coated porous SnO_2_ film detected 0.5 ppm at room temperature but required 184/432 s response/recovery [38].

Interface control can amplify the SnO_2_ response without relying solely on larger surface area. One-step CVD growth of ZnO nanorods directly on a SnO_2_ film simplified heterojunction fabrication and yielded a 370% response to 50 ppm at 300 °C over a 0.05–1000 ppm test range [97]. More broadly, the ALD-based sensor literature shows how conformal nanoscale coatings can tune heterointerface thickness and depletion behavior, whereas direct CVD routes favor simpler batch-compatible integration [98,99]. This contrast highlights a recurring manufacturing trade-off between interfacial precision and process throughput.

Controlled oxidation of tin sulfides creates oxide–sulfide junctions with lower activation requirements than pristine SnO_2_. Partial oxidation of SnS_2_ produced SnO_2_/SnS_2_ hollow microspheres with stronger adsorption and higher response [62], while time-resolved oxidation of ultrathin SnS_2_ showed that SnO_2_ nucleates first at nanoflower edges and then spreads across basal regions, establishing an optimum interfacial fraction at 60 °C [65]. SnS_2_-rGO improves conductivity and reversibility at 80 °C but still exhibits approximately 6 min response and 53 min recovery at 10 ppm [86]. Earlier SnO_2_@ZnO hierarchical heterostructures provide a complementary oxide–oxide comparison for interface-amplified NO_2_ sensing [100].

Post-oxidized SnO_x_/SnS extends this strategy to room-temperature trace sensing. It reached a 171% response at 1 ppb, the lowest experimentally delivered concentration in that study, while UV exposure shortened recovery to 36 s [101]. The result demonstrates the combined roles of Schottky contacts, oxide–sulfide charge transfer, oxygen vacancies, and active regeneration without relying on extrapolated trace metrics for cross-study ranking.

Two-dimensional conductors address aggregation and charge-transport limitations. SnO_2_/MXene suppressed nanoparticle agglomeration, increased accessible area, and created a work-function-modulated heterojunction, producing a 231% response to 30 ppb at room temperature with 146/102 s kinetics [58]. Dielectrophoretically assembled CNT/SnO_2_ [52] linked the composition ratio to the junction density and detected 20 ppb, while LIG/SnO_2_ [102] offered direct patterning on polyimide but showed gas trapping and incomplete recovery. SnO_2_ therefore spans high-performance precision interfaces and low-cost printed platforms, with recovery and humidity remaining the principal discriminators.

**Figure 5:**
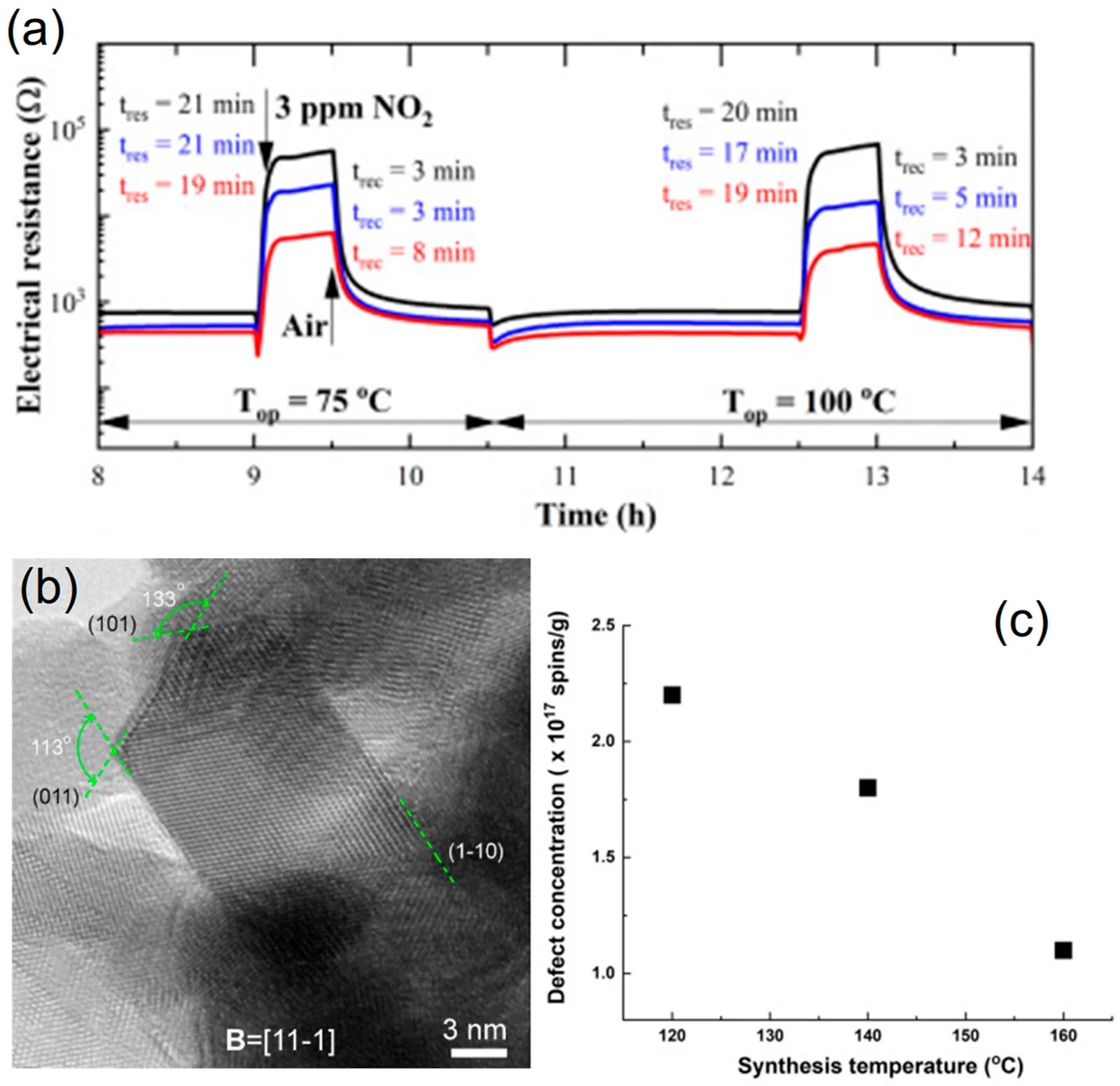
Correlation between NO_2_ sensing performance and structural/defect characteristics of SnO_2_ nanocrystals. (**a**) Electrical-resistance transients to 3 ppm NO_2_ at 75 and 100 °C. (**b**) HRTEM image showing exposed crystal facets. (**c**) EPR-derived concentration of surface oxygen-vacancy-related defects. All panels adapted with permission from Ref. [25].

Figure 5 links the electrical sensing trend to independent structural and spectroscopic evidence. The SnO_2_ sample synthesized at 120 °C gives the strongest transient response, HRTEM identifies exposed crystal facets, and EPR shows that the concentration of surface oxygen-vacancy-related paramagnetic defects decreases with increasing synthesis temperature. Together, these data support an association between surface faceting, vacancy-related defects, and the observed NO_2_ sensing trend rather than inferring the mechanism from electrical response alone [25].

### 4.3. WO_3_: Defect/Dopant Engineering, Catalytic Sensitization, and Functional Interfaces

WO_3_ has strong affinity for oxidizing gases but commonly requires moderate heating because the same adsorption strength that supports sensitivity can hinder desorption. Y-doped WO_3_ nanoplates [68] used rare-earth substitution to manipulate oxygen vacancies and produced a 94-fold enhancement relative to pure WO_3_, with 7/38 s response/recovery at 150 °C. Ag-WO_3_ [76] combined vacancy formation, catalytic spillover, and work-function modification, reporting a 52,404% response to 1000 ppb and 1/3.5 s kinetics at the same temperature. Because this value is reported using the source paper’s percentage-response convention, it is retained without renormalization and is not treated as directly rankable against ratio-based WO_3_ metrics from other studies.

Cobalt doping demonstrates that defect engineering can target recovery as well as response. The optimized 3 mM% Co-WO_3_ nanoplates produced a 20,776% response to 10 ppm at 200 °C with 15/23 s response/recovery, compared with 26/154 s for pristine WO_3_, and retained approximately 95% response over eight weeks [72]. The proposed Co^3+^/Co^2+^ redox mediation and interstitial lattice distortion increase oxygen-vacancy density, but the useful outcome is kinetic: the dopant reduces the recovery penalty that often accompanies strong NO_2_ binding.

Organic surface chemistry offers a different route. APTES-functionalized WO_3_-x nanowires under UV light showed about 20-fold higher NO_2_ sensitivity than unmodified WO_3_-x because terminal amino groups facilitate interaction and charge transfer [85]. However, the sensor required a 9 min response at 10 ppm, high UV irradiance, and an up to 25 min purge for complete recovery. Functional-group affinity must therefore be evaluated against optical power, response time, desorption completeness, and long-term silane stability.

WO_3_ also benefits from transducer and mixed-dimensional engineering. Flexible WO_3_/MoS_2_ reached 364.5% at 1 ppm and 10 ppb testing at 125 °C before integration with an OLED readout and user-defined buzzer [11]. A WO_3_ horizontal-floating-gate FET used charge-storage engineering to increase response by 2.4 times and sensitivity by 3.2 times [103]. These platforms show that WO_3_ performance can be improved by controlling the receptor, the junction, and the electrostatic readout simultaneously.

**Figure 6:**
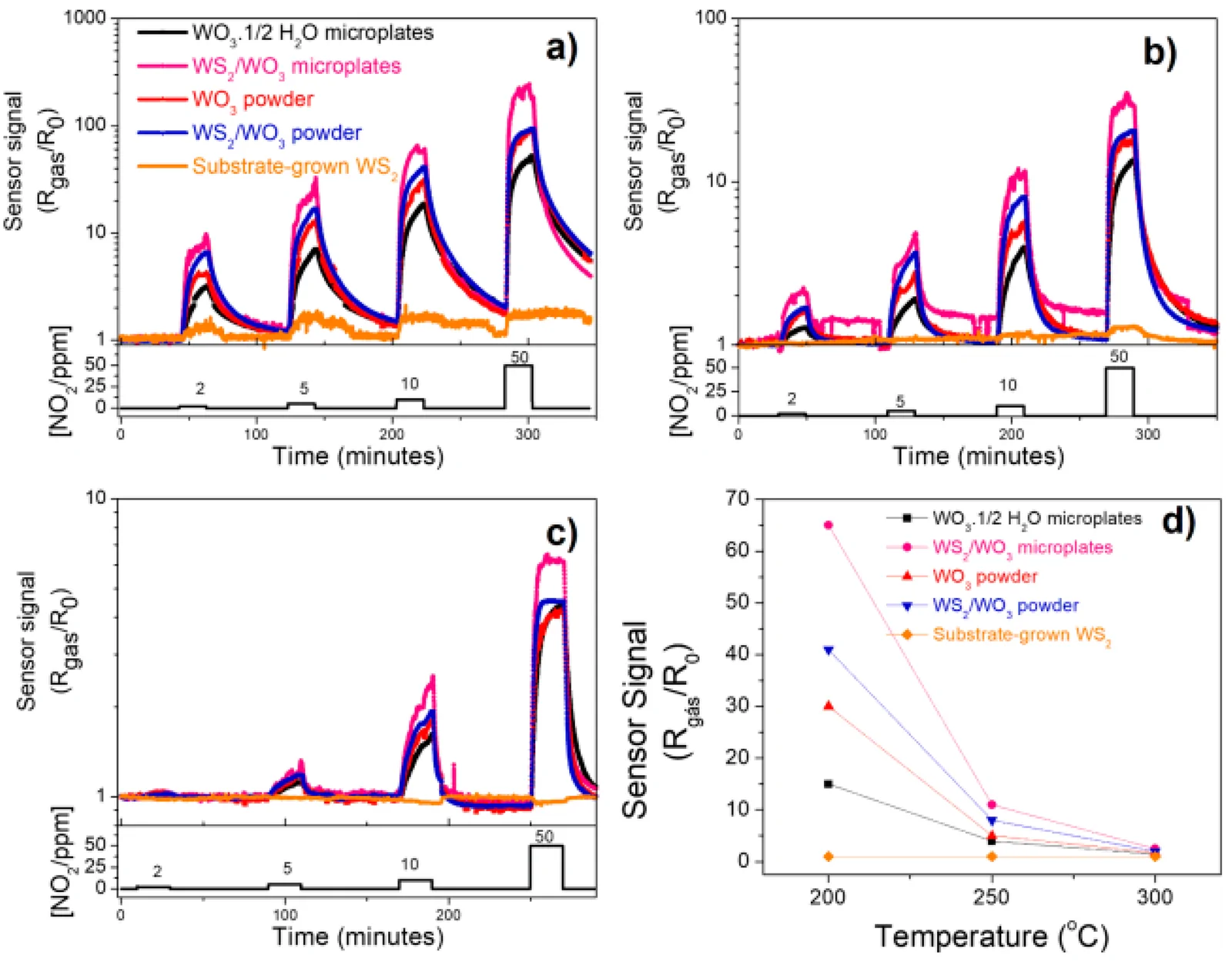
Transient sensor signal for NO_2_ exposure at different temperatures for the WO_3_ seed materials and WO_3_/WS_2_ heterostructures: (**a**) 200 °C; (**b**) 250 °C; (**c**) 300 °C. (**d**) Maximum sensor signal measured for 10 ppm exposure at each working temperature. All panels adapted with permission from Ref. [44].

Figure 6 isolates the contribution of the WS_2_/WO_3_ junction by comparing each heterostructure with its tungsten-oxide seed material at identical temperatures. All compositions show the expected oxidizing-gas tendency of increasing resistance, but the WS_2_/WO_3_ microplate heterostructure produces the largest signal at 200 °C, and the enhancement weakens as temperature is raised to 250–300 °C. The authors attribute this behavior to electronic sensitization across the oxide/sulfide junction. Formation of the junction introduces an interfacial potential barrier; NO_2_-induced charge withdrawal changes the carrier populations on both sides and increases the effective barrier, so the interface converts a surface adsorption event into a larger conductance modulation. Figure 6 therefore highlights both the junction benefit and the temperature window required to preserve it [44].

### 4.4. In_2_O_3_: Phase/Defect Engineering, Heterointerfaces, and Active Regeneration

In_2_O_3_ combines a wide band gap, relatively high conductivity, and strong compatibility with thin-film and porous processing. In_2_O_3_-In_2_(MoO_4_)_3_ [104] provides a wide 0.2–150 ppm range and survives a 10,000 ppm concentration shock. TiO_2_ quantum dots on In_2_O_3_ nanotubes [69] increase oxygen deficiency and yield a response of 214.3 to 10 ppm at 120 °C. Pristine In_2_O_3_ nanobricks calcined at 400 °C remain competitive, giving a response of 402 to 500 ppb at 50 °C with 114/49 s kinetics and testing to 100 ppb [105].

Heterostructures tune carrier transfer and selectivity. Fe_2_O_3_/In_2_O_3_ composites provide dual-selective operation, with the optimized FIO-3 sensor reaching 64.3 at 700 ppb NO_2_ and responding at 10 ppb [63]. In_2_S_3_/In_2_O_3_ nanoflowers use calcination time to control phase fraction and interface area [64]. In_2_O_3_ nanoparticles on rough GaN produced Rg/Ra = 1070.9 at 1 ppm and 100 °C with experimental 1 ppb detection [106], while Au@In_2_S_3_/In_2_O_3_ hybrid microflowers provide a room-temperature noble-metal-assisted In_2_O_3_ example [77].

Phase and regeneration control are equally important. Visible-light-regenerated In_2_O_3_ nanowires retained a room-temperature response of 740 at 5 ppm under dark conditions while shortening recovery to about 20 s [41]. Thermally oxidized 100 nm In_2_O_3_ films provided dual NO_2_/H_2_S detection at 27 °C with 58% NO_2_ response at 5 ppm and experimental detection to 100 ppb, although recovery at 10 ppm remained longer than 1500 s [107]. MOF-In_2_O_3–_400 combined mixed cubic/hexagonal phases, high oxygen-vacancy content, and porous morphology to reach a response of 1210 at 200 ppb and detect 1 ppb at 30 °C with pulse-heated recovery [73].

Flexible and two-dimensional composites broaden the deployment space. UV-activated In_2_O_3_/MXene showed a response of 27.65 to 50 ppb, linear behavior from 5 to 100 ppb, more than 2000 bending cycles, and tolerance to 70% RH and direct water exposure [88]. Coaxially electrospun rGO@In_2_O_3_ nanofibers on PET reached a response of 14.18 at 1 ppm and were experimentally tested to 100 ppb while retaining bending tolerance [108]. The g-C_3_N_4_/In_2_O_3_ array [15] further couples material diversity with CNN-AGRU analysis of partial transients. In_2_O_3_-graphene-Cu nanocomposites provide another example in which carbon-assisted transport and metallic modification are used to tune selectivity and low-concentration response [109].

Across these studies, In_2_O_3_ performance depends on more than peak response. Mixed phases and vacancies can improve sensitivity, but humidity, overload recovery, pulse energy, baseline resistance, and interface reproducibility determine whether the material can move from a high-response chamber result to a calibrated sensor node.

The vertically stacked In_2_O_3_/In_2_S_3_ study [45] illustrates the interfacial origin of the room-temperature gain in this heterostructure. Prior to contact, the two semiconductors have different band positions; after self-assembly, Fermi-level equilibration creates a type-II alignment and a built-in field across the van der Waals interface. This field redistributes carriers and makes the device conductance highly sensitive to further electron withdrawal by adsorbed NO_2_. In_2_O_3_ contributes reactive adsorption sites, including vacancy-associated sites, whereas In_2_S_3_ provides a complementary transport and adsorption pathway; the vertical 2D/2D geometry maximizes the electronically coupled interface. The adsorption schematic therefore connects the enhanced response with interface-mediated charge separation and with adsorption–desorption dynamics that can be assisted by optical excitation [45].

### 4.5. Other Metal Oxides and Wide-Bandgap Semiconductor Platforms

CuO-based p-type systems provide a complementary response polarity and strong compatibility with rGO and TMD interfaces. CuO/rGO [50] operates at room temperature with a 6.8 s response, while a face-to-face porous CuO/rGO architecture reached 1004% at 5 ppm and 30 °C with a 9 s response, although recovery required 110 s [110]. Under red-light illumination, CuO nanospheres on MoS_2_ produced a response of 8.98 to 10 ppm at room temperature with 18.5/53.5 s kinetics and testing to 50 ppb [89]. Hydrangea-like CuO assembled from porous nanosheets reached Ra/Rg = 133.6 at 10 ppm and 50 °C with a 4 s response, but recovery required 452 s [111].

CuO/CeO_2_ [59] uses a p-n interface and ceria oxygen storage to enhance low-temperature NO_2_ response at 100 °C. CuO/TiO_2_ nanorod arrays [61] show room-temperature selectivity, although repeated cycling reduced response, emphasizing that a high initial signal does not guarantee reproducibility. Vertical CuO nanowires integrated on MEMS [112] demonstrate the importance of controlled heating and IC-compatible fabrication, while hierarchical Cu_2_O-CuO microflowers combine mixed valence, adsorbed oxygen, and 3D assembly to detect 5 ppb at 187 °C with 35/47 s kinetics [113].

Other p-type and redox-active oxides broaden the operating envelope. A porous sprayed NiO film produced 57.3% sensitivity to 20 ppm at 200 °C, with deposition time controlling the transition from honeycomb porosity to denser films [114]. CeO_2_ nanoparticles showed temperature-dependent selectivity between H_2_S and NO_2_, linking response to crystallite size, Ce^3+^/Ce^4+^ balance, and oxygen vacancies [74]. Au nanoparticles on approximately 6 nm Co_3_O_4_ particles integrated on a MEMS heater produced a 33% response to 100 ppb at 136 °C, benefiting from a nanoscale area, Au spillover, and a metal–semiconductor junction [79]. MOF-derived Co_3_O_4_/SiW_12_ illustrates porous POM-mediated sensing but has multi-minute complete cycles [10].

GaN and Ga_2_O_3_ offer chemical, thermal, and mechanical robustness. KOH-etched p-GaN hexagonal pits detected 2 ppm at room temperature with a 6.2% response and 41/33 s kinetics; UV exposure increased the 100 ppm response by about 1.3 times [115]. A graphene channel coupled to undoped GaN nanowires reached 23% at 100 ppm under 100 mW cm^−2^ xenon illumination and retained 95% of its initial response after six months, although the lowest tested concentration was 10 ppm [116]. Hollow, porous GaN nanofibers achieved a response of 22.9 at 200 ppm, 11/105 s kinetics, and a reported 0.5 ppb limit at 200 °C [75].

Gold-black nanoparticles on Ga_2_O_3_ nanorods provide a wide-bandgap p-n/spillover route: the optimum 60 s decoration yielded 5221.1% at 10 ppm and 56.9% at 100 ppb at 230 °C with 27.3/69.2 s kinetics [80]. These wide-bandgap systems favor harsh-environment stability and catalyst compatibility, but their operating temperature, concentration range, and optical power must be matched to the intended application.

### 4.6. Carbon-Based Materials and Hybrid Networks: rGO, CNTs, and Laser-Induced Graphene

Carbon materials contribute high carrier mobility, low-temperature conductivity, mechanical flexibility, and abundant defect sites. rGO is most effective as an interfacial or percolating phase rather than as a standalone receptor [18,34,35,47,50,53,54]. In hybrid films, it can accelerate transport, prevent oxide aggregation, and enable flexible substrates, but loading must be controlled to avoid shunting the oxide response or shielding adsorption sites.

The one-step dielectrophoretic CNT/SnO_2_ study [52] directly links composition ratio to p-n junction density. In artificial air, the optimized sensor showed a normalized response of about 19 to 1 ppm and was experimentally tested at 20 ppb, while UV illumination was used to manage oxygen competition. The long acquisition required at trace concentration illustrates why the lowest tested concentration should be accompanied by response time, drift, and recovery fraction rather than treated as a standalone performance label.

The stacked rGO/ZnO nanowire work [92] demonstrates that carbon distribution is as important as content. Spin-casting controlled mono-to-few-layer rGO bridges avoided agglomeration and created a more uniform built-in potential than conventional drop coating. Finite-difference simulations connected layer count with local potential and charge-transfer pathways, providing a device-scale link between interface geometry and local transport.

Laser-induced graphene [102] offers an attractive manufacturing route because porous conductive carbon can be patterned directly on polyimide. LIG and LIG/SnO_2_ responded at room temperature and were repeatable at 50 ppm, but response times of roughly 435–475 s and incomplete recovery reveal gas trapping in the porous network. The platform is promising for low-cost flexible fabrication, but pore architecture and regeneration require further optimization.

Carbon-assisted oxide sensors now include receptor modification and active thermal control. A one-pot room-temperature CuO/rGO synthesis produced a response of about 14 at 1 ppm with 66/34 s kinetics [117]. A self-supporting rGO/α-Fe_2_O_3_ architecture grown directly on ITO reached R_g_/R_a_ = 32.42 at 100 ppm and improved response more than eightfold over α-Fe_2_O_3_, with practical testing to 0.3 ppm [118]. In a device-level variant, a transparent CNT receptor integrated with a 175 nm SnO_2_ memristor heater gave 19.42% at 0.5 ppm and pulse-assisted recovery while maintaining heater performance over 50 cycles [13].

The N-rGO/NiO study [27] illustrates why combining p-type NiO with N-rGO can produce a much larger response than either component alone. In air, adsorbed oxygen removes electrons from NiO and forms a near-surface hole-accumulation layer. When NO_2_ is introduced, additional electron withdrawal further increases the hole population and changes the thickness of this conducting surface region, lowering the resistance of the p-type network. N-rGO contributes nitrogen-containing adsorption sites and a high-mobility percolation pathway, while the p–p interface redistributes charge between NiO and the carbon sheet. The resulting sensor therefore couples surface redox chemistry, accumulation-layer modulation, and interfacial transport, explaining why the hybrid can exhibit very large low-concentration responses relative to bare N-rGO [27].

### 4.7. Two-Dimensional Materials and Mixed-Dimensional Heterostructures: TMDs and MXenes

TMDs provide a high surface-to-volume ratio and tunable electronic structure, but pristine materials often recover slowly. Co-incorporated MoS_2_ [119] increases active edge sites and improves response at room temperature, yet recovery remains on the order of minutes. SnS_2_ [120] was evaluated at 100 °C over ppm-scale NO_2_ exposure, while partial oxidation to SnO_2_/SnS_2_ hollow microspheres [62] increases response through both morphology and an oxide–sulfide heterojunction. Broader TMD and van der Waals sensing studies likewise emphasize layer-dependent adsorption, Schottky contacts, and hybrid charge-transfer pathways [23,121,122,123].

MXenes combine metallic conductivity with surface terminations that support adsorption and heterostructure formation [48,51,55,56,57]. In_2_O_3_/Ti_3_C_2_ [48] and Ti_3_C_2_Tx@ZnO [55] use Schottky barriers and vacancy-rich oxide surfaces. The cotton-modified LaFeO_3_/MXene composite [51] is notable for its room-temperature response of 14.2 to 5 ppm, 2.7/6.2 s kinetics, and a wireless IoT leakage prototype.

Hierarchical hollow MoS_2_ microspheres exposed more edge sites and improved gas transport, producing an 88.3% response to 500 ppm at 150 °C and measurable response at 500 ppb, although recovery remained slow [124]. PW_12_-modified SnS_2_ lowered the optimum temperature to 200 °C and gave 24/30 s kinetics [84]. CuO/Ti_3_C_2_Tx operated at room temperature with a 15.6% response to 10 ppm, but its approximately 500 s response time shows that high conductivity and abundant vacancies do not automatically solve diffusion-limited kinetics [125].

Three 2D-device studies couple interface engineering with optical or transistor control. UV-activated flexible In_2_O_3_/MXene showed linear 5–100 ppb behavior together with mechanical and water tolerance [88]. Microwave-assisted Au functionalization of CVD monolayer MoS_2_ produced an approximately 10% current response to 200 ppb during a 15 s exposure and UV-assisted reset within a few seconds [81]. SnO_2_/MXene [58] provides the chemiresistive counterpart, where the conducting phase improves charge transport and prevents oxide aggregation.

The PTCDI/Ti_3_C_2_T_x_ MXene study [46] extends heterojunction sensing to an organic–inorganic van der Waals interface. When PTCDI and Ti_3_C_2_T_x_ MXene contact each other, charge transfer associated with their different electronic levels creates an interfacial field and modifies the transport barrier. Under blue-light excitation, photogenerated carriers are separated across this interface, which increases the population of mobile carriers and changes the baseline barrier. NO_2_ subsequently acts as an electron acceptor, depleting electrons from the PTCDI-rich transport channel and increasing the effective space-charge/barrier modulation, thereby producing a large resistance response. The schematic also emphasizes the need for an optimum MXene fraction: sufficient interface density is beneficial for charge separation, whereas excessive conductive phase can reduce the accessible adsorption area or shunt the gas-sensitive pathway [46].

Few-layer GaSe showed a −94.5% response to 2 ppm and linear behavior from 80 ppb to 2 ppm, but its optimum temperature was 200 °C [126]. Machine learning-assisted material selection identified Ga_2_O_3_/MoS_2_ as a sputter-compatible heterostructure; the device detected 100 ppb at 25 °C, and 365 nm UV exposure increased the response while shortening recovery [7]. Flexible WO_3_/MoS_2_ reached 10 ppb testing at 125 °C and progressed to an OLED readout and buzzer alarm [11].

Nitrogen doping and Ag decoration provide a dual surface-engineering strategy for MoS_2_. Ag-N-MoS_2_ reached a 71.02% response to 50 ppm at 100 °C with 27.17/132.24 s response/recovery, outperforming pristine, Ag-only, and N-only controls; the device was also evaluated over 11 weeks and 20–80% RH [82]. The enhancement is mechanistically useful, but the 1–50 ppm range is oriented more toward occupational or industrial monitoring than ambient ppb exposure.

### 4.8. Porous, MOF-Derived, and Template-Engineered Architectures

MOF-derived oxides inherit high porosity and can generate mixed-metal junctions during pyrolysis. Porous ZnO derived from ZIF-8 [36] exposes connected gas pathways, while NiO-SnO_2_ derived from a bimetallic MOF [49] combines a 74.73 m^2^/g surface area, oxygen vacancies, and a p-n junction for 25 ppb detection at room temperature and 60% RH. The approach is powerful, but solvent use, calcination energy, batch yield, and preservation of pore architecture should be reported for scale-up.

Biomimetic and sacrificial-template methods offer related advantages. The dewdrop-like carbon template [40] creates hierarchical rough layers in situ, and cotton-modified LaFeO_3_ [51] uses a low-cost natural scaffold to generate porosity. These strategies move beyond maximizing BET area in powders by considering how the active network is electrically connected to electrodes.

Recent papers show how porous architecture can be combined with junction control. Bimetallic-MOF-derived ZnO/NiO operated at room temperature under UV light, giving a response of 6.78 at 1 ppm, 15/40 s kinetics, and experimental detection to 20 ppb [9]. Self-supporting rGO/α-Fe_2_O_3_ [118] avoids slurry binders, while gold-black/Ga_2_O_3_ [80] demonstrates that optimum catalyst coverage balances spillover against blocked adsorption area. Hydrangea-like CuO [111] shows that a high-area porous structure can accelerate adsorption without guaranteeing equally rapid recovery.

### 4.9. Conducting Polymers and Mechanically Compliant Organic Semiconductors

Polyaniline, polypyrrole, PEDOT, and related conducting polymers can respond to electron-accepting NO_2_ near room temperature, offering low-temperature processing and intrinsic mechanical compliance [1]. Their principal limitations are humidity-dependent doping, solvent or vapor swelling, baseline drift, and batch-dependent morphology. Polymer sensors should therefore be benchmarked with the same rigor as oxides, including dynamic RH, recovery completeness, strain history, and aging.

The nanoporous P3HT@SEBS sensor [127] demonstrates that a stretchable organic semiconductor can combine gas access with mechanical stress dispersion. Breath-figure porosity produced an approximately 4842% response at 10 ppm and retention of more than 89% normalized responsivity at 40% tensile strain. The result is important for wearable design, although incomplete room-temperature recovery remains a central limitation.

**Figure 7:**
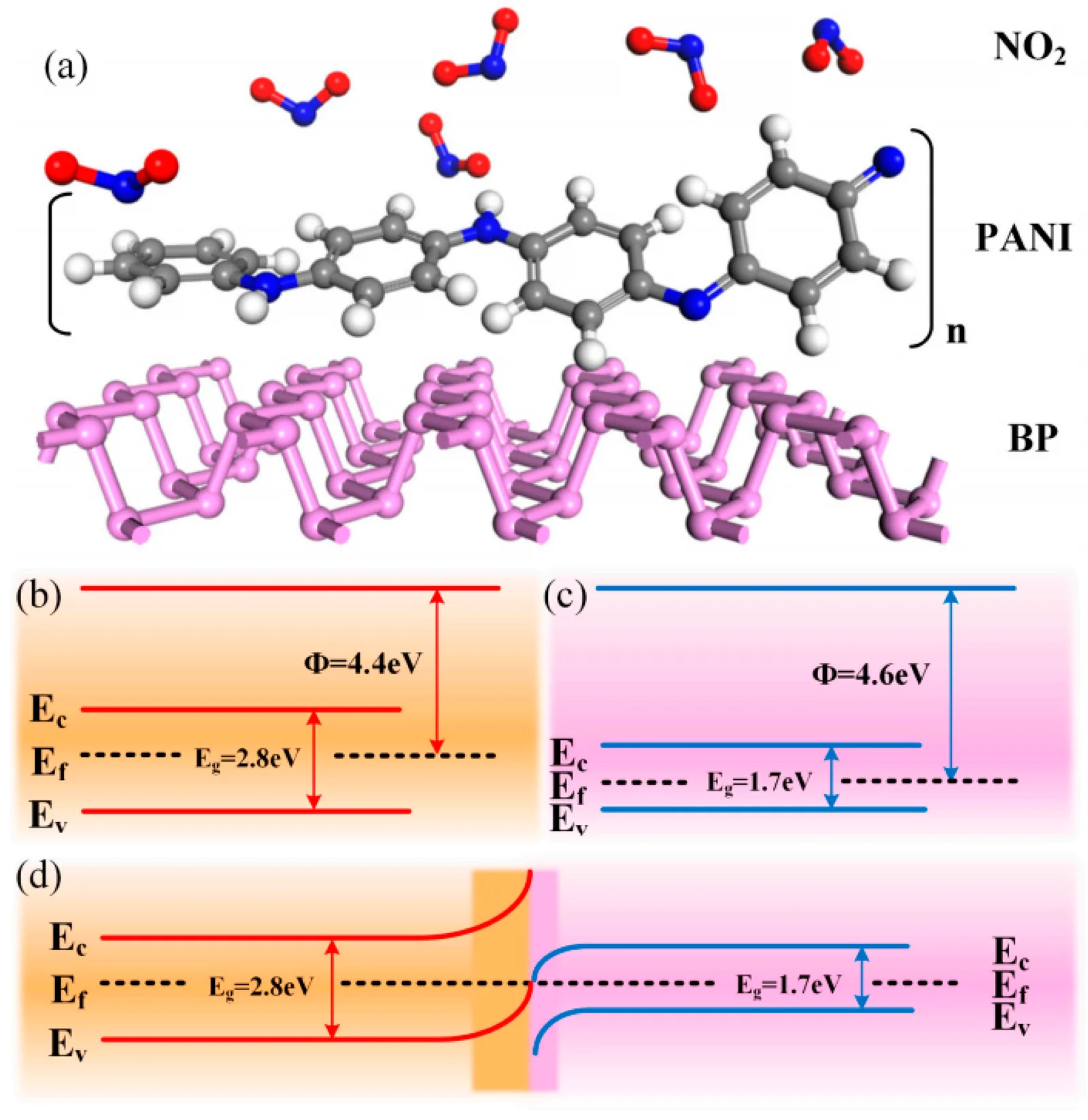
(**a**) Molecular schematic of NO_2_ gas adsorption by polyaniline (PANI)/black phosphorus (BP); band diagram of (**b**) PANI, (**c**) BP, and (**d**) PANI/BP. All panels adapted with permission from Ref. [28].

Figure 7 explains how the PANI/BP composite addresses both sensitivity and baseline drift at room temperature. PANI helps separate the originally thick BP crystallites into more exposed, thinner flakes, increasing the fraction of BP that participates in surface charge transfer. At the same time, contact between PANI and BP forms a p/p-type junction because their Fermi levels differ; carrier redistribution at this interface provides an additional barrier-sensitive conduction pathway. This interface is particularly important for stability because NO_2_ can strongly oxidize PANI and leave a persistent conductance shift when PANI is used alone. Incorporating BP alters the interfacial carrier balance and substantially reduces this zero-drift behavior while retaining strong NO_2_ response, so Figure 7 links the molecular adsorption picture directly to the composite band alignment [28].

Table 2 summarizes the material-platform discussion by linking the dominant engineering routes to their main advantages and unresolved trade-offs.

## 5. Device Architectures, Transduction, and Integration Strategies

### 5.1. Flexible, Transparent, and Directly Patterned Sensor Platforms

The flexible WO_3_/MoS_2_ study [11] provides a useful distinction between mechanical flexibility and thermal operation. The PI-based device maintained its baseline and response through bending and 200 cycles, yet its strongest result occurred near 125 °C. Mechanical compliance therefore should not be interpreted as proof of room-temperature or battery-compatible operation unless heater power and thermal isolation are reported.

Transparent MoS_2_/rGO adds optical transparency as a device constraint. The patterned film retained approximately 93% transmittance on PET and sensing characteristics under bending while detecting 0.15 ppm at 90 °C [128]. This platform shows that transparent wearable sensors require simultaneous reporting of sheet resistance, optical transmittance, bending radius, cycle count, gas response, and regeneration energy.

The new flexible platforms emphasize different mechanical design philosophies. In_2_O_3_/MXene uses a conductive 2D scaffold on PDMS and preserves its response after more than 2000 bending cycles and direct water exposure [88]. The breath-figure P3HT@SEBS sensor transfers a nanoporous organic film onto a stretchable substrate and serpentine Au electrodes, retaining sensing under 40% strain [127]. CdTe/ZnO@porous-Si [66] and one-step CVD ZnO/SnO_2_ [97] are rigid devices, but their direct thin-film routes are compatible with planar integration and batch processing.

Flexible integration is strengthened by the PET-supported rGO@In_2_O_3_ nanofiber sensor [108], which retained reversible operation under 30–120° bending, and by the Smart Breath Sentinel [14], which packaged an In(OH)_3_-α-Fe_2_O_3_-ZnO sensor with temperature/humidity measurement, wireless transmission, and a mask-mounted housing. The latter is not a passive mask filter; it is a personal environmental warning platform whose accuracy depends on sensor heating, humidity compensation, and circuit calibration.

Planar interdigitated electrodes remain the most common transducer because they are simple, scalable, and compatible with drop-casting, screen printing, spin coating, spraying, and direct writing. The flexible rGO/SnO_2_ warning device [96], Au@In_2_S_3_/In_2_O_3_ on PET [77], LIG/SnO_2_ on polyimide [102], and ZnO/rGO [34] demonstrate different routes to room-temperature operation. For flexible devices, sensing performance should be reported as a function of bending radius, cycle count, strain direction, and encapsulation, not just before and after a single bending test.

**Figure 8:**
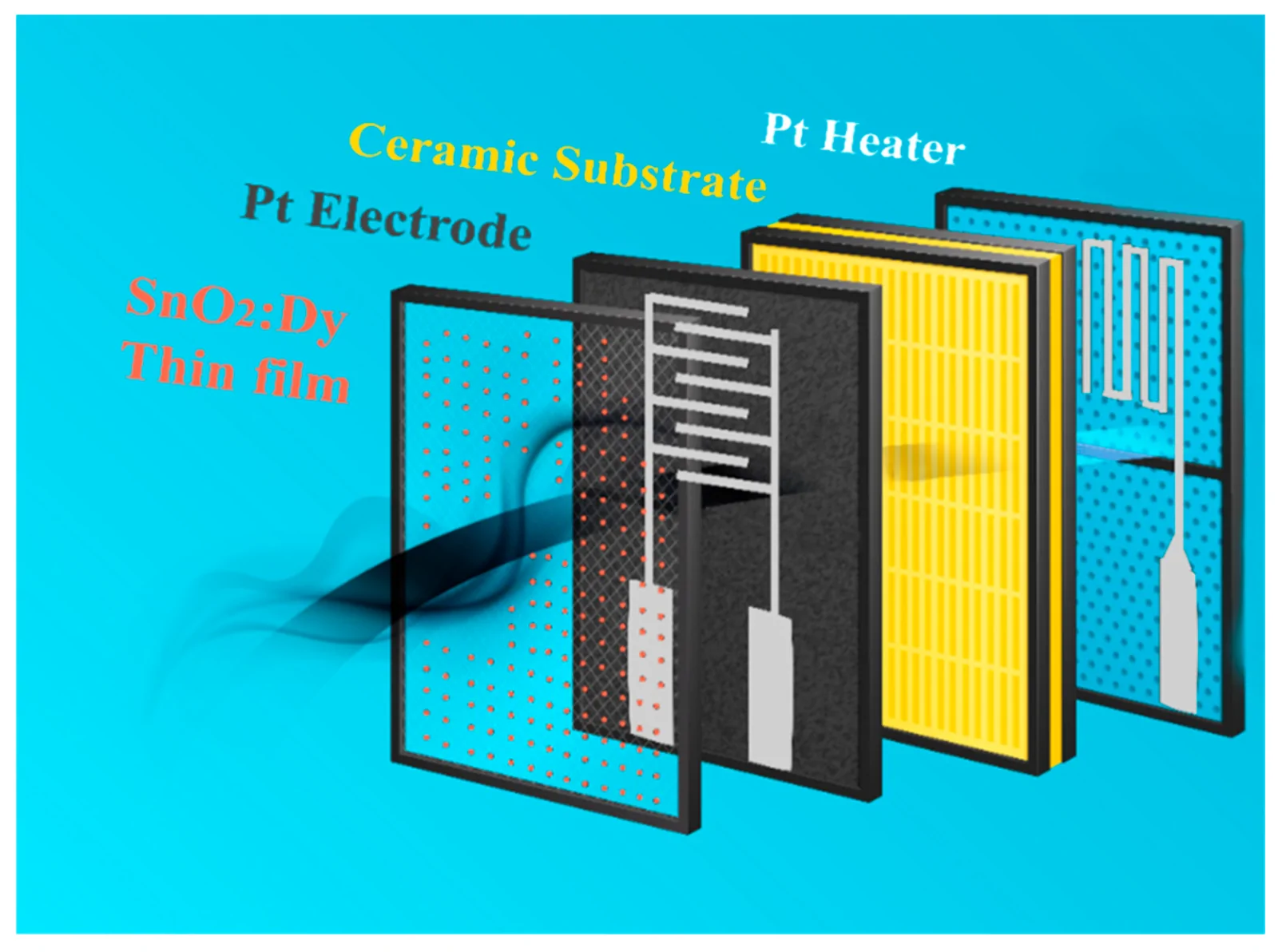
Schematic drawing of the R.F.-sputtered SnO_2_:Dy thin film sensor structure deposited on alumina substrates. All panels adapted with permission from Ref. [26].

Figure 8 is a useful device-level reference for interpreting the SnO_2_:Dy material results because it separates the sensing film, electrical readout, and thermal actuation. The RF-sputtered SnO_2_:Dy layer covers a ceramic alumina platform with Pt electrodes for resistance measurement and a Pt heater for controlled operation from room temperature to elevated temperature. The authors selected this substrate specifically because it allows both film resistance and operating temperature to be set reproducibly. This architecture made it possible to compare Dy contents under the same electrical and thermal geometry and showed an important trade-off: measurable room-temperature NO_2_ response was obtained, but the signals were less stable than those recorded at a higher temperature. Thus, the figure connects dopant engineering to the practical need for controlled thermal transduction [26].

### 5.2. MEMS Microheaters and Localized Thermal Actuation

MEMS platforms reduce heater mass and enable precise temperature modulation. The sputtered CuO nanowire/MEMS sensor [112] operates at 119 °C and demonstrates stable ppb-level response with low long-term inaccuracy. The Si FET sensor with a localized In_2_O_3_ microheater [129] detects 25 ppb while the heater consumes approximately 0.84 mW at a 2 V, 50% duty-cycle condition. Local thermal isolation and pulsed operation make this approach more relevant to battery-powered nodes than a nominal operating temperature alone suggests.

The Au/Co_3_O_4_ nanoparticle/MEMS device [79] extends this concept to a p-type receptor. Its 33% response to 100 ppb at 136 °C is lower in nominal magnitude than some thick-film oxides, but the small sensing area, integrated heater, 10 ppb testing, and reproducible thermal control are more relevant to compact IoT nodes than response magnitude alone.

The SnO_2_ memristor-heater/CNT structure [13] extends localized heating beyond conventional resistors. The heater thickness controlled conductive-filament formation, temperature, response, and variability; 175 nm SnO_2_ gave the best trade-off and was more durable than the prior ZnO heater over repeated cycles. Because the sensing film remained a room-temperature CNT network while recovery was pulse-heated, this architecture should be benchmarked by energy per pulse, recovery fraction, and heater-cycle endurance rather than ambient temperature alone.

**Figure 9:**
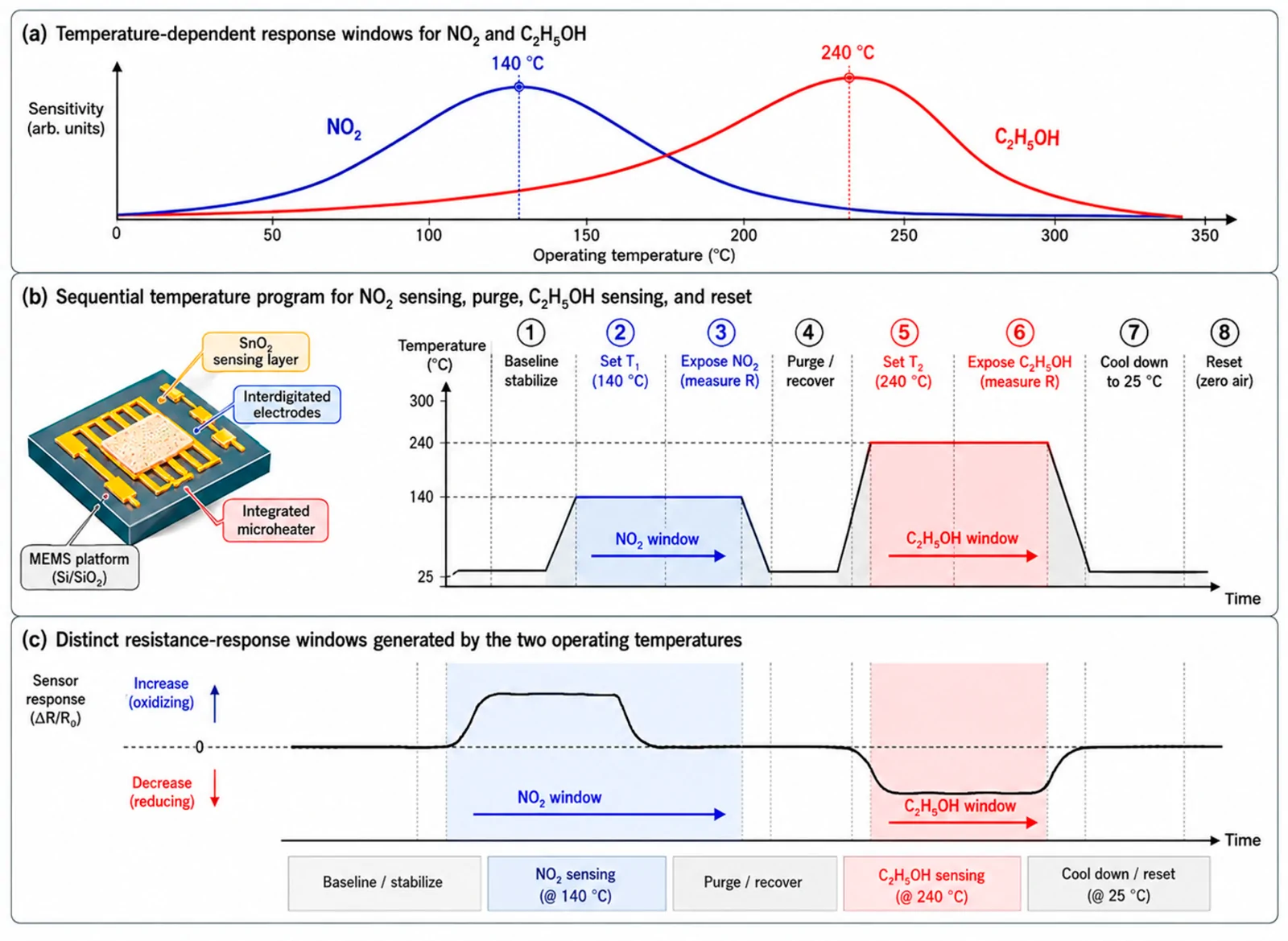
Temperature-programmed sequential sensing using a SnO_2_ MEMS sensor. (**a**) Schematic temperature-dependent response windows for NO_2_ and C_2_H_5_OH. (**b**) Sequential temperature program for NO_2_ sensing, purge, C_2_H_5_OH sensing, and reset. (**c**) Schematic resistance-response windows generated by the two operating temperatures. This schematic was prepared with the assistance of ChatGPT (OpenAI) and was subsequently reviewed, edited, and verified by the author.

Figure 9 schematically illustrates the temperature-programmed sequential sensing strategy. The integrated microheater switches the SnO_2_ sensing element between the NO_2_-sensitive state at approximately 140 °C and the C_2_H_5_OH-sensitive state at approximately 240 °C, with purge and reset steps between measurements. This temperature modulation enables a single sensing element to generate gas-dependent temporal response windows. [87].

**Figure 10:**
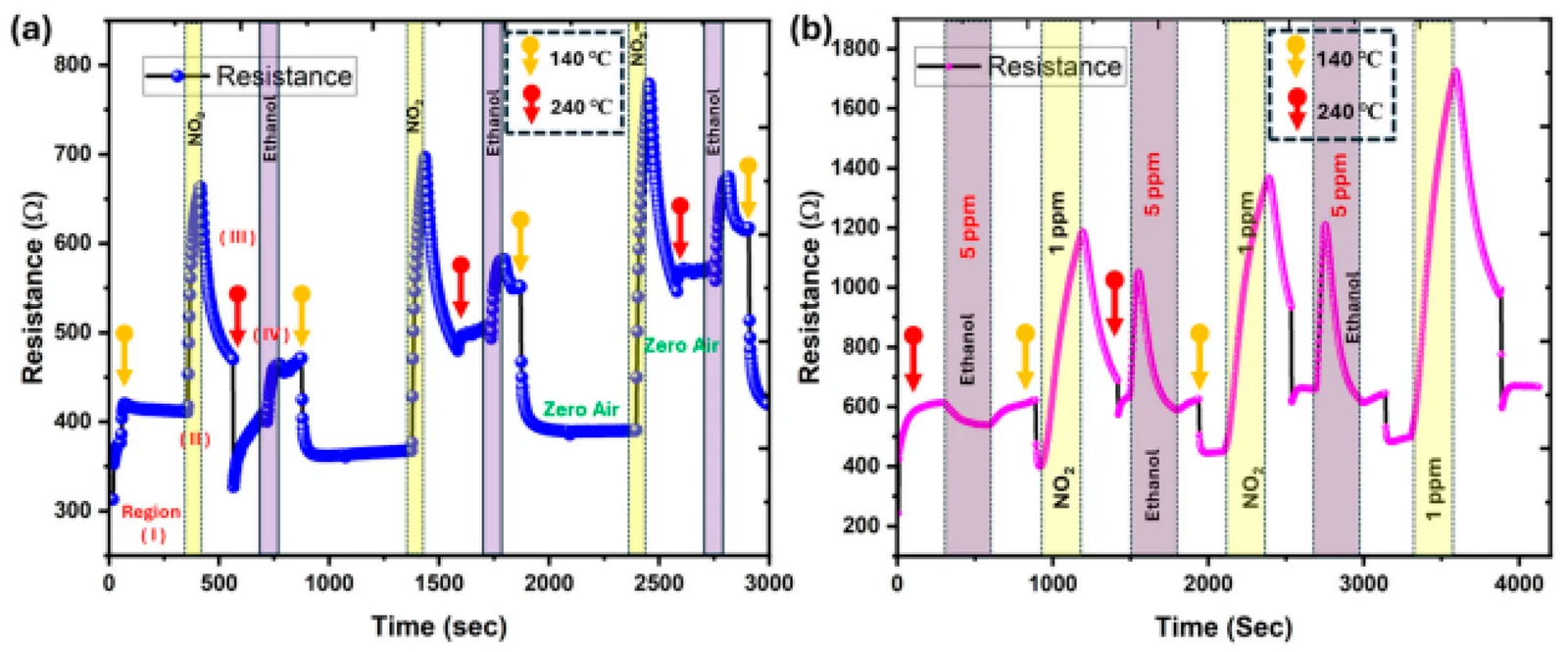
Experimental sequential sensing of NO_2_ and C_2_H_5_OH using the SnO_2_ MEMS sensor. (**a**) Sequential response to NO_2_ followed by C_2_H_5_OH at their respective operating temperatures. (**b**) Response for the reverse exposure sequence, C_2_H_5_OH followed by NO_2_. All panels adapted with permission from Ref. [87].

Figure 10 provides the experimental demonstration of this concept. Sequential exposure to NO_2_ and C_2_H_5_OH at their respective operating temperatures produces distinct resistance responses, while the reverse exposure sequence further reveals the influence of exposure history and surface recovery [87].

### 5.3. FET/TFT/MOSFET Transduction and Electrical Control

FET and TFT transducers separate the conductive channel from the gas-sensitive surface and provide additional electrical degrees of freedom. In the WO_3_ floating-gate device [103], charge-storage engineering altered carrier density near the sensing interface, increasing response by 2.4 times and sensitivity by 3.2 times. In the In_2_O_3_ TFT [90], the floating-gate state enhanced response at 75 °C, and a pulsed negative gate bias desorbed NO_2_^−^ rapidly, enabling recovery within one second.

Au-functionalized monolayer MoS_2_ adds a FET-specific readout dimension: both current response and threshold-voltage shift were monitored during 15 s, 200 ppb exposures at 30 °C [81]. Microwave absorption enabled low-temperature, short-time nanoparticle functionalization, and UV illumination restored the transfer curve within a few seconds. This study should be compared by bias, transconductance, exposure time, and recovery protocol rather than by resistance ratio alone.

Low-temperature performance depends on transducer engineering as well as receptor optimization. A useful comparison is energy per complete measurement cycle, including warm-up, adsorption, readout, regeneration, and wireless transmission; reporting only heater power or describing a receptor as room-temperature can understate the energy required for recovery and communication.

A co-sputtered zinc indium tin oxide (Zn-In-Sn-O, ZITO) sensing layer integrated with a horizontal-floating-gate MOSFET demonstrates composition-transducer co-optimization [12]. Oxygen-vacancy-rich ZITO produced the highest response near 210 °C, whereas a hydroxy-rich composition performed better near 120–150 °C and reduced heater demand. The study included 500 ppb testing, but response and recovery depend strongly on composition, microheater temperature, and control-gate bias; these variables should be reported together.

**Figure 11:**
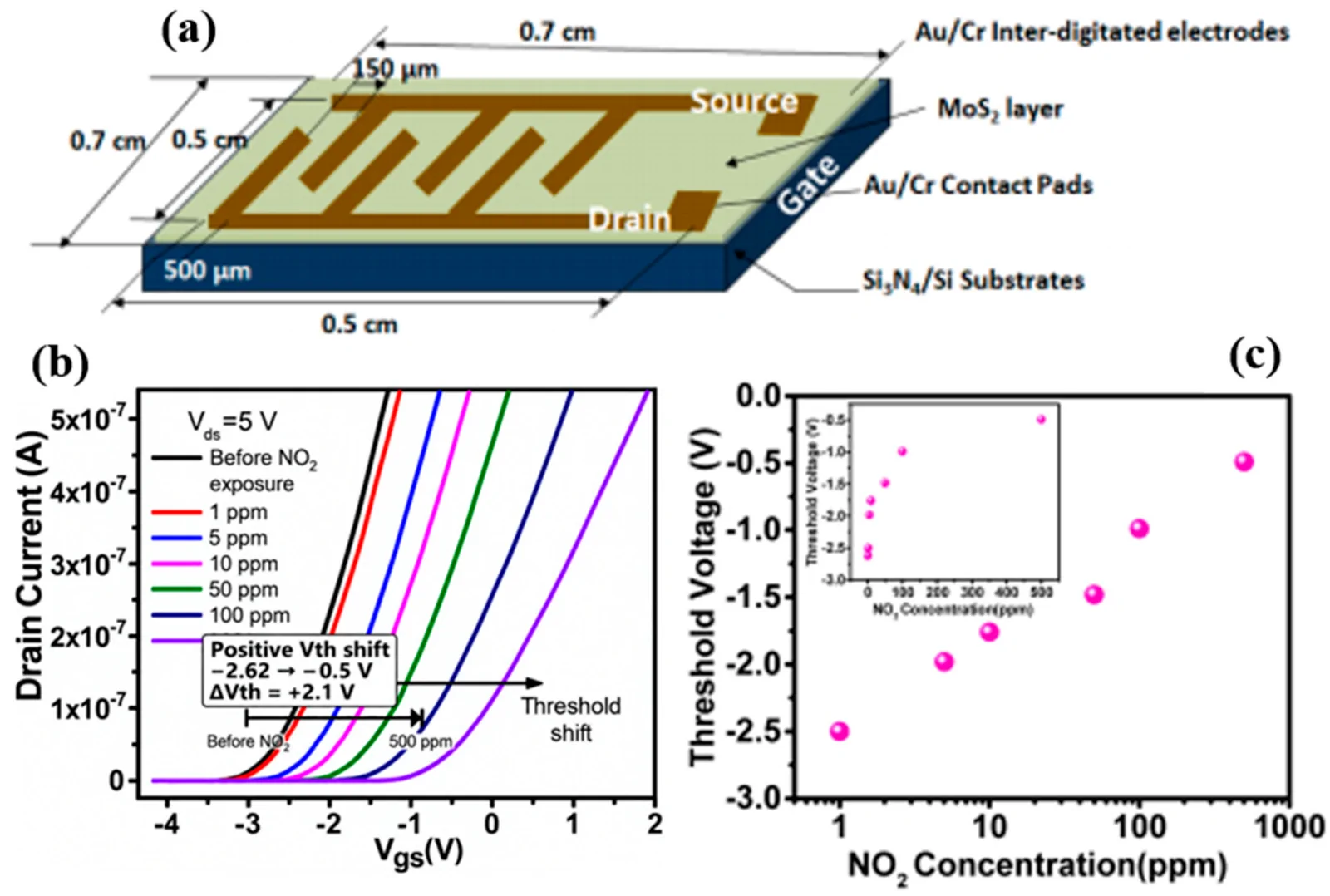
NO_2_ sensing characteristics of a back-gated MoS_2_ FET. (**a**) Schematic of the MoS_2_ FET sensor structure. (**b**) Transfer characteristics showing a positive threshold-voltage shift with increasing NO_2_ concentration. (**c**) Threshold voltage as a function of NO_2_ concentration. All panels adapted with permission from Ref. [29].

Figure 11 illustrates the additional information available when NO_2_ adsorption is read through a field-effect transistor rather than a two-terminal resistor. Increasing the NO_2_ concentration strongly suppresses the drain current of the MoS_2_ channel and produces a systematic shift of the transfer characteristic, consistent with electron withdrawal from an n-type MoS_2_ conduction channel. The gate electrode therefore converts adsorbate-induced surface charge into both a current-amplitude change and a threshold-voltage shift, providing two related transduction observables. The four-layer device showed the strongest overall sensing response among the layer counts examined, indicating a practical balance between abundant exposed surface, continuous carrier transport, and electrostatic gate control. Consequently, the curves in Figure 11 demonstrate how gate modulation can amplify and interrogate room-temperature NO_2_ charge transfer [29].

### 5.4. Photoactivation and Hybrid Regeneration Strategies

UV illumination can generate carriers, remove adsorbates, or reset the baseline. CNT/SnO_2_ [52] uses UV light to reduce oxygen interference, while rough-layer ZnO [40] uses a short UV pulse specifically for recovery. The distinction matters: continuous illumination changes the sensing mechanism and power budget, whereas duty-cycled regeneration preserves low baseline power. Similar adaptive strategies can combine room-temperature adsorption with brief thermal, optical, or electrical pulses only when recovery is incomplete. Monolithic micro-LED/metal-oxide concepts further show how optical activation can be integrated at microwatt-class device scale [91].

UV-assisted Ga_2_O_3_/MoS_2_ [7] illustrates a balanced regeneration strategy in air: at 100 ppb and 25 °C, 365 nm illumination increased the response from 0.48% to 1.484% and reduced the response time to 40 s and the recovery time from 67 to 51 s. The study also combined a humidity/temperature sensor with an optimized Kalman-filter edge terminal, showing how optical activation and signal stabilization can be co-designed.

Visible-light-regenerated In_2_O_3_ nanowires provide a particularly clear adsorption-reset separation [41]. The sensor retained a high dark response at room temperature, while a recovery-only visible-light pulse reduced recovery from about 1000 s to 20 s at an optimized intensity. Excess intensity produced a transient photocurrent and apparent recovery before complete desorption, demonstrating why optical reset protocols should distinguish electrical baseline restoration from molecular removal.

The UV-activated In_2_O_3_/MXene sensor [88] and red-light CuO/MoS_2_ sensor [89] show two additional activation modes. In_2_O_3_/MXene used continuous UV light to enhance both adsorption and desorption and maintained nearly complete recovery, whereas CuO/MoS_2_ used red light to improve charge separation across a p-n interface. The light wavelength, intensity, duty cycle, and photo-current baseline must therefore be reported as part of the operating condition, not treated as an auxiliary detail.

Photoactivation is not universally benign; its benefits and long-term stability depend on receptor composition, catalyst state, wavelength, intensity, duty cycle, and cumulative photon dose. Ag-ZnO responses degraded with accumulated operation, especially under UV light, because crystal quality, vacancy density, Ag oxidation, dark conductivity, and photoconductivity evolved together [17]. APTES@WO_3_-x [85] required high UV irradiance and long response/purge periods, whereas other systems use visible light or short recovery-only pulses. Photoactivation should therefore be benchmarked together with source aging, dark resistance, recovery completeness, and post-illumination sensitivity.

**Figure 12:**
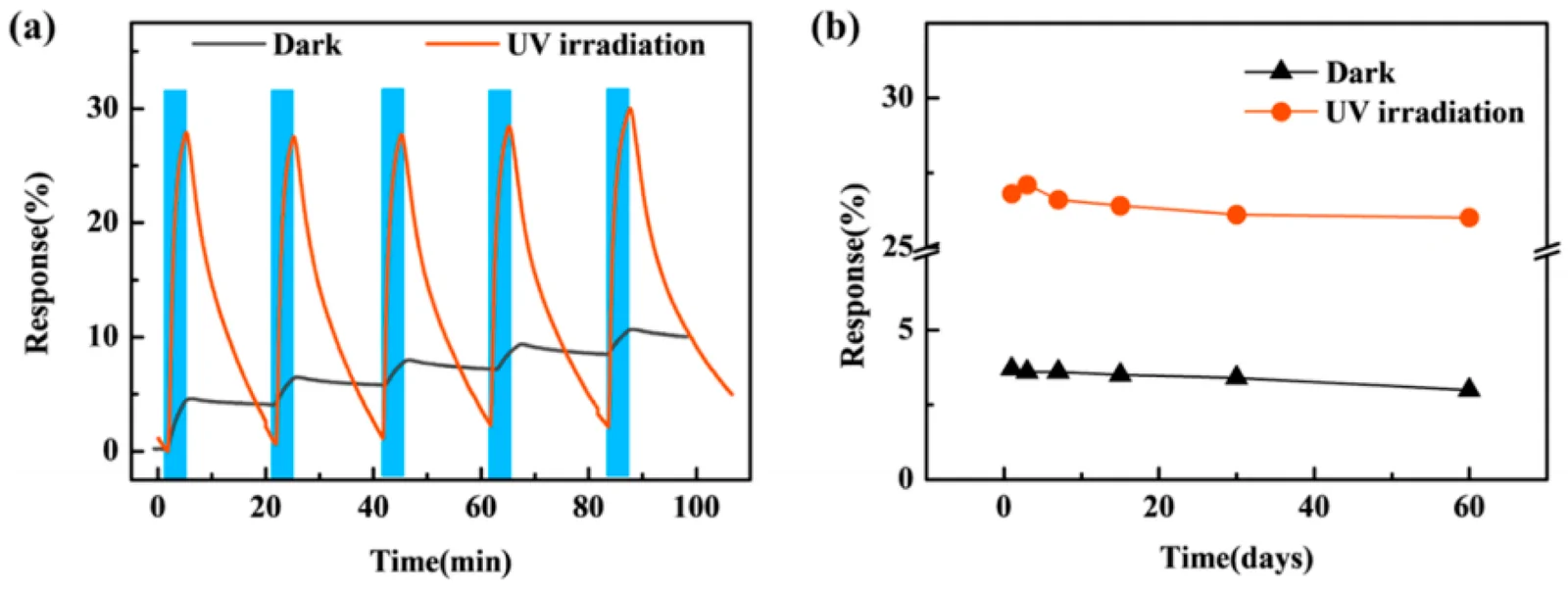
Comparison of dark and UV-assisted operation of the graphene NO_2_ sensor: (**a**) repeatability and (**b**) long-term stability at room temperature. All panels adapted with permission from Ref. [30].

Figure 12 was reduced to the two source-paper panels that most directly compare dark and UV-assisted operation. The repeated exposure data show stronger and more reproducible NO_2_ response under UV light, while the long-term comparison highlights that optical activation must be evaluated together with stability rather than treated as a universally beneficial reset. The figure therefore supports treating illumination as a controlled operating variable whose short-term recovery benefit must be balanced against cumulative optical exposure [30].

### 5.5. Photoacoustic and Spectroscopic Transduction

Chemiresistors infer concentration from surface-induced electrical changes, whereas photoacoustic spectroscopy measures acoustic pressure generated by optical absorption and nonradiative relaxation. The dual-modality photoacoustic cell [3] excited Helmholtz and longitudinal resonances in one chamber to detect NO and NO_2_ simultaneously, with tens-of-ppb sensitivity after extended integration and 35.7/32.5 s response/recovery. These metrics are not directly comparable with resistance ratios because optical power, modulation depth, resonator quality factor, sample flow, and averaging time determine the usable concentration resolution. The study is nevertheless valuable as a reference for applications requiring NO/NO_2_ speciation and low cross-sensitivity.

**Figure 13:**
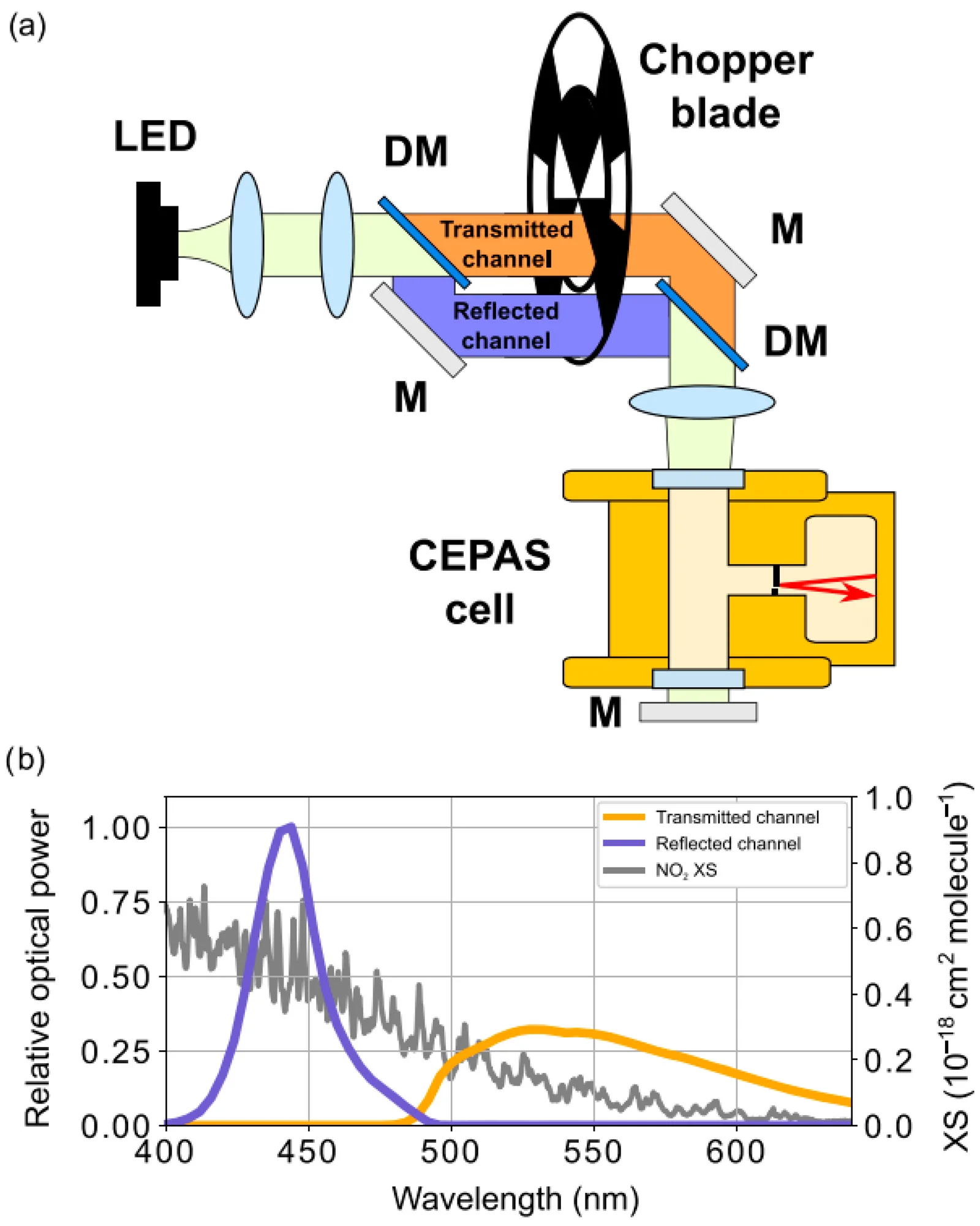
(**a**) Schematic of the two-channel NO_2_ concentration measurement setup. DM: dichroic beamsplitter; M: aluminum mirror. The red arrow denotes the cantilever optical readout system. (**b**) Relative LED optical power spectra in the two wavelength branches, derived from the LED spectrum and dichroic-mirror characteristics reported in Ref. [130]. All panels adapted with permission from Ref. [130].

Figure 13 represents a fundamentally different NO_2_ transduction route that does not rely on adsorption-induced conductivity. A broadband white LED is divided by dichroic optics into two wavelength channels with different NO_2_ absorption strengths, and both beams are modulated and measured simultaneously in a cantilever-enhanced photoacoustic cell. Light absorbed by NO_2_ is converted through nonradiative relaxation into an acoustic signal, whereas absorption or scattering at the cell walls contributes a common background to both channels. Taking the relative two-channel response suppresses this correlated background and associated drift while retaining the spectrally selective NO_2_ contribution. This differential optical architecture is the key reason the LED-based system can reach sub-ppb noise levels after averaging without the high-cost laser source normally used for trace photoacoustic spectroscopy [130].

### 5.6. AI-Assisted Sensing, IoT Integration, and Distributed Monitoring

Signal processing and connectivity are part of the sensing problem, but so is reproducibility of the physical input. Machine learning can compensate humidity and temperature, classify gas mixtures, detect drift, transfer calibration across devices, extract concentration from partial transients, and guide heterostructure selection [1,7,15]. The flow rate study [16] shows a central risk: a model can learn chamber hydrodynamics or a particular device baseline unless total flow, exchange time, day, and device identity are controlled or included as explicit variables.

**Figure 14:**
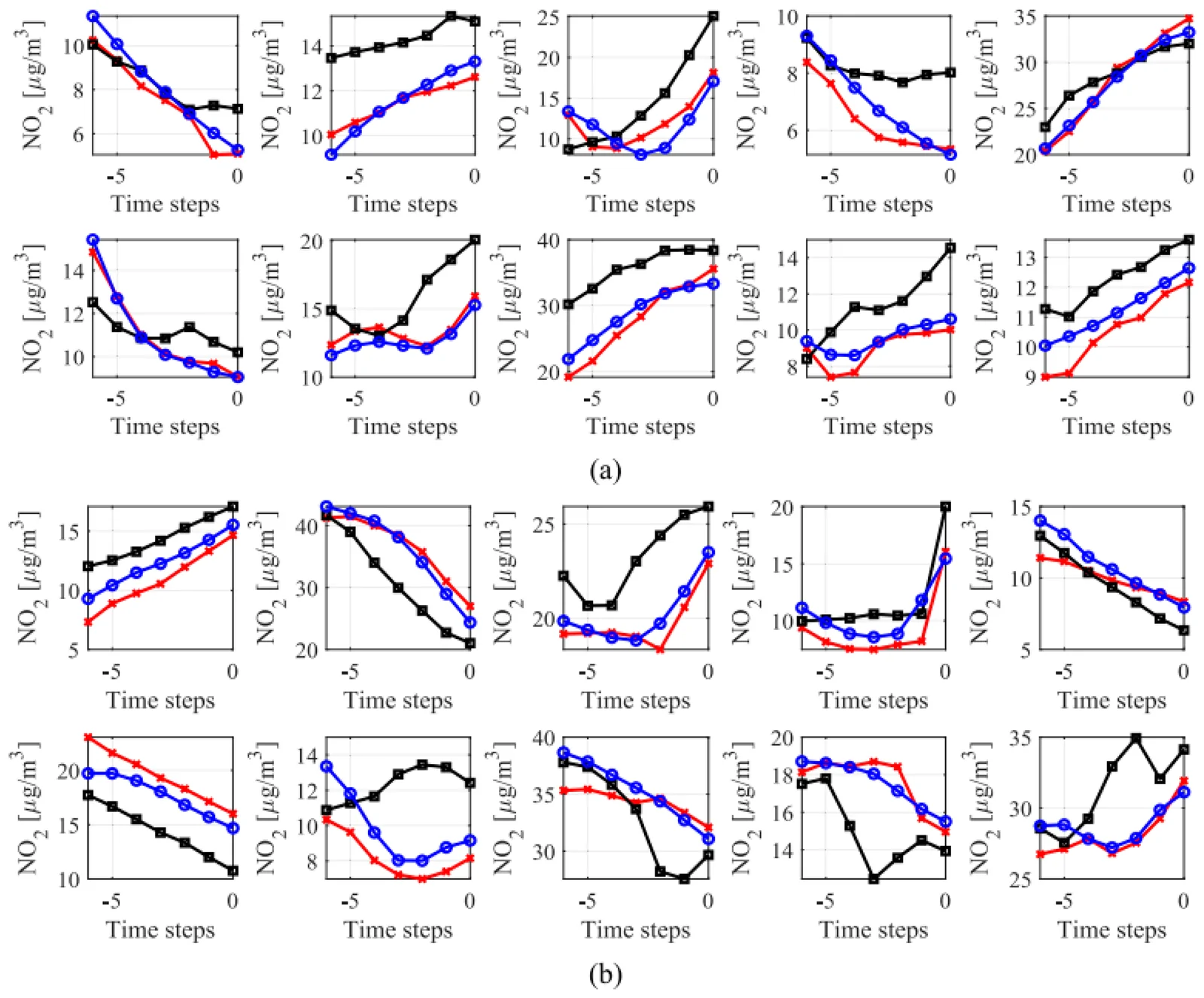
Time series alignment obtained using the ANN surrogate for Nt = 6. Shown are the raw (uncorrected) low-cost sensor data (black), reference time series (red), and ANN-predicted sensor time series (blue). Significant improvement of the data alignment can be observed: (**a**) selected training points; (**b**) selected testing points. All panels adapted with permission from Ref. [131].

Figure 14 demonstrates that field calibration should be viewed as a dynamic mapping problem rather than as a single offset or slope correction. The uncorrected low-cost NO_2_ signal (black) departs visibly from the reference-station series (red), whereas the ANN prediction (blue) follows both the amplitude and temporal evolution much more closely in the training and validation intervals. The calibration model uses not only the primary sensor value but also temperature, humidity, atmospheric pressure, redundant NO_2_ channels, and a short history of previous samples, allowing it to learn environmental and time-dependent distortions. Together with global statistical preprocessing and an auxiliary interpolation model, this approach produced a reported correlation close to 0.95 and an RMSE of 2.4 µg m^−3^. Figure 14 therefore illustrates why algorithms and environmental metadata are part of the measurement chain for distributed low-cost NO_2_ sensing [131].

The MXene/CLFO study [51] demonstrates a wireless leakage prototype, the flexible rGO/SnO_2_ work [96] integrates an over-limit warning node into a wearable format, and flexible WO_3_/MoS_2_ [11] adds continuous OLED display with user-defined buzzer thresholds. The Smart Breath Sentinel [14] combines a heated In(OH)_3_-α-Fe_2_O_3_-ZnO receptor, temperature/humidity sensing, an STM32 controller, Wi-Fi transmission, and a mask-mounted enclosure. Ga_2_O_3_/MoS_2_ [7] uses machine learning band-alignment screening and an OBL-QPSO-UKF edge filter, while the g-C_3_N_4_/In_2_O_3_ array [15] uses CNN-AGRU to predict 1–9 ppm NO_2_ from partial transients.

A deployable data-driven system should separate four layers of performance: receptor response, analog front-end transfer, algorithmic inference, and communication or alarm logic. Validation should use device- and day-separated train/test sets, external reference concentrations, dynamic RH and flow, calibration transfer to unseen devices, uncertainty estimates, and out-of-distribution detection. Otherwise, improved prediction accuracy may reflect memorization of a chamber, a batch, or a preprocessing pipeline rather than robust chemical selectivity.

The device-level strategies in Section 5 are summarized in Table 3, emphasizing that architecture determines not only signal magnitude but also power, recovery, mechanical integration, and calibration requirements.

## 6. Environmental Effects, Selectivity, and Long-Term Reliability

### 6.1. Humidity Effects: Competitive Adsorption, Charge Transport, and Compensation

Water affects NO_2_ sensing through several coupled pathways. Molecular water competes with NO_2_ and oxygen for adsorption sites, changes surface hydroxyl coverage, alters protonic and electronic conduction, and can modify the effective work function or junction barrier. In porous films, humidity also changes diffusion and capillary retention; in rGO and polymers, it can dope the conductive network or cause swelling. The direction and magnitude of the humidity effect therefore depend on carrier type, temperature, surface termination, and whether response is limited by adsorption, charge transfer, or transport. Room-temperature operation should therefore be treated separately from ambient-humidity robustness; a low-temperature signal alone does not establish stable field operation.

The reviewed studies illustrate this diversity. Flexible rGO@In_2_O_3_ loses response as RH increases [108], co-sputtered ZITO responses decline from 3.5% to 55% RH [12], and self-supporting rGO/α-Fe_2_O_3_ decreases above approximately 54% RH [118]. By contrast, Ag-N-MoS_2_ remains operational from 20% to 80% RH [82], the SnO_2_-heater/CNT device still reports a 27.67% response at 90% RH [13], and UV-activated In_2_O_3_/MXene retains function at 70% RH and after direct water exposure [88]. These results are not directly rankable because concentration, temperature, response definition, and stabilization time differ.

Humidity resilience should be designed rather than corrected after fabrication. Useful strategies include hydrophobic but gas-permeable membranes, selective organosilane or catalytic surface chemistry, differential reference channels, local pulse heating to desorb water, and co-located RH/temperature sensors. The Smart Breath Sentinel [14] demonstrates explicit humidity compensation at the system level, but a credible protocol should train and validate correction under dynamic RH ramps, abrupt condensation events, and day-to-day device drift rather than a small set of fixed humidity points.

Static RH tolerance should not be conflated with dynamic humidity robustness. A sensor may retain a measurable NO_2_ response at a fixed humidity yet show a large baseline transient when RH changes rapidly or when condensation occurs. This distinction is especially important for flexible, wearable, and breath-adjacent systems. Future studies should therefore report not only steady-state response-versus-RH curves but also transient concentration error during RH steps, baseline re-equilibration time, and recovery after near-condensation conditions.

**Figure 15:**
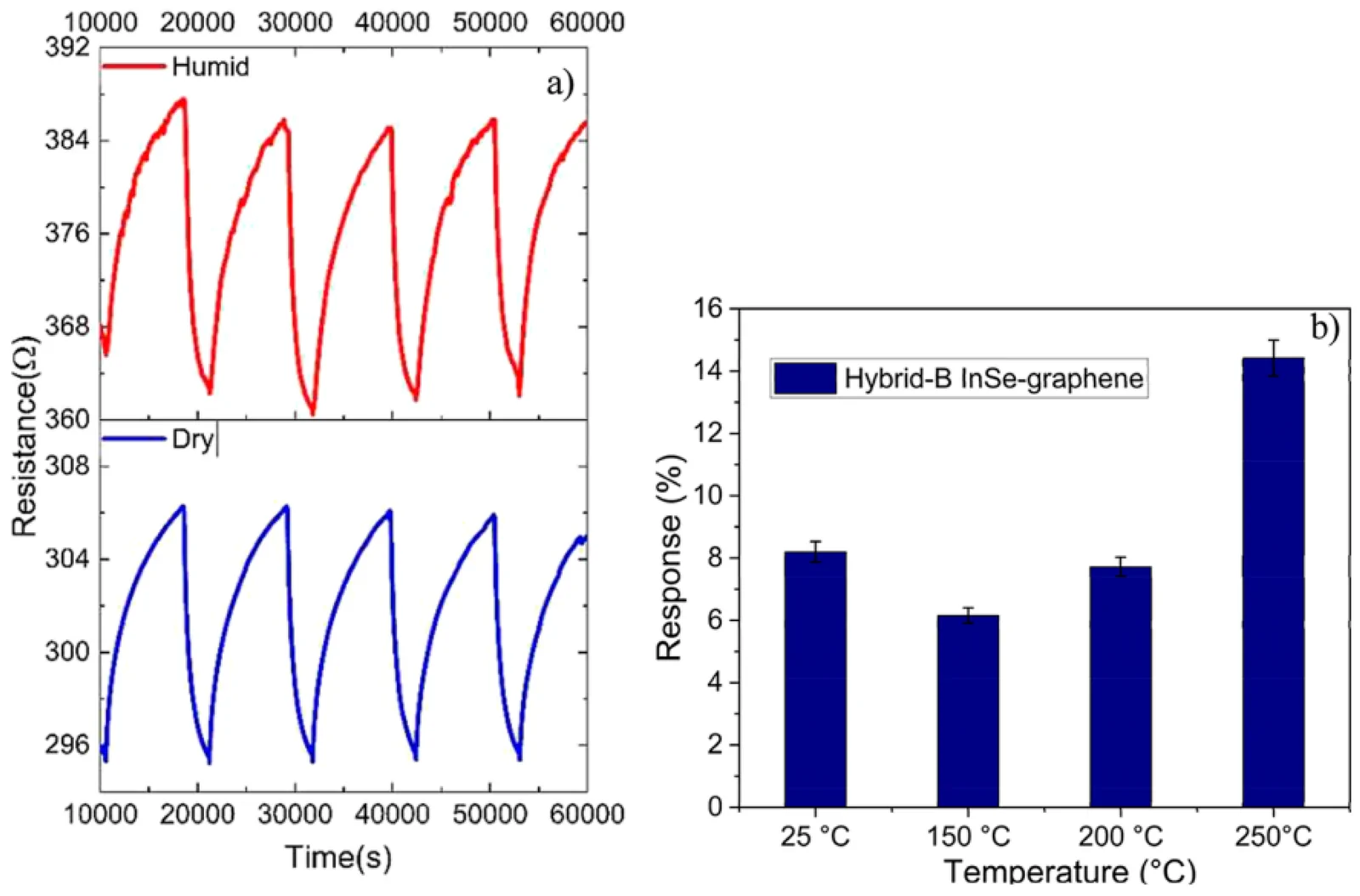
(**a**) Comparison of hybrid B InSe–graphene sensor response under dry and humid conditions. Sensor operated at 150 °C. (**b**) Hybrid-B sensor responses at different operating temperatures under humid conditions. All panels adapted with permission from Ref. [31].

Figure 15 provides an important counterexample to the common assumption that humidity always suppresses NO_2_ response. For the hybrid InSe–graphene sensor, 50% RH increased the response relative to dry operation. The authors relate this behavior to oxygenated defects on the exfoliated graphene and to water-derived terminal and bridging hydroxyl groups, which create additional surface chemistry that favors NO_2_ adsorption and nitrite formation. Under humid conditions, response also increased markedly at 250 °C; at that temperature, further conversion of nitrite-type surface species toward nitrate-type species may contribute. The result emphasizes that humidity can either block sites or create reactive intermediates depending on receptor chemistry, so RH effects must be measured mechanistically rather than treated as a universal negative correction [31].

### 6.2. Selectivity Under Interferents and Realistic Gas-Mixture Conditions

Selectivity can be intrinsic, operational, or computational. Intrinsic selectivity arises from adsorption energy, acid–base chemistry, catalytic dissociation, pore size, and defect chemistry. Operational selectivity uses temperature, gate bias, illumination wavelength, or pulsed heating to create a response pattern. Computational selectivity combines arrays or time-dependent features with multivariate models. A strong selectivity claim should identify which layer provides discrimination and whether the mechanism remains valid when gases are mixed.

Temperature-dependent dual selectivity is demonstrated by In_2_O_3_ thin films for NO_2_/H_2_S [107], MOF-derived In_2_O_3_ for NO_2_/TEA [73], CeO_2_ nanoparticles for H_2_S versus NO_2_ [74], and Fe_2_O_3_/In_2_O_3_ composites at different operating temperatures [63]. Optical enhancement in CuO/MoS_2_ [89], surface functionalization in Au@In_2_S_3_/In_2_O_3_ [77], and bias-controlled FET platforms [12,90,103] show that illumination, interface chemistry, and electrical state can also act as selectivity dimensions. However, changing temperature or bias simultaneously changes baseline resistance, oxygen speciation, kinetics, and noise, so cross-gas calibration must be repeated at every operating state.

**Figure 16:**
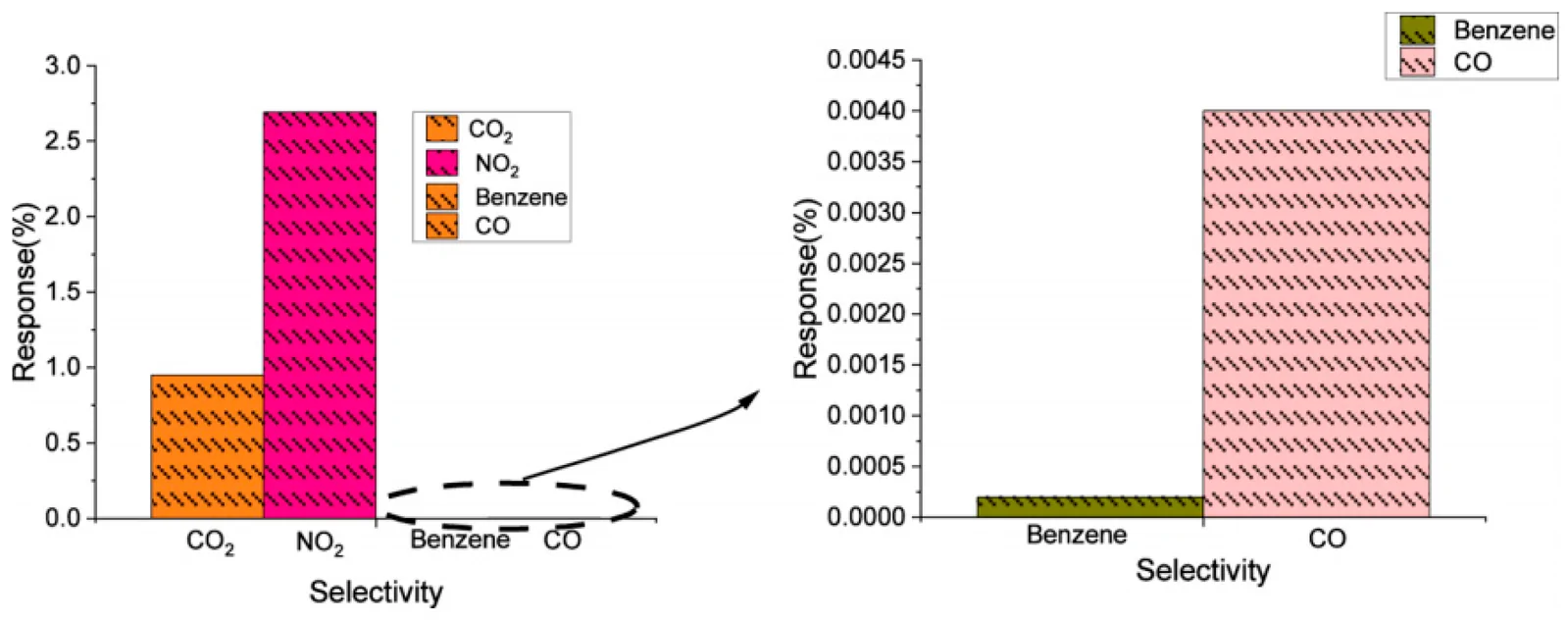
Selectivity test for different gases. Sensor operated at room temperature under dry conditions. All panels adapted with permission from Ref. [31].

Figure 16 shows a larger NO_2_ response than the tested CO, CO_2_, and benzene interferents under the reported dry, room-temperature conditions. This result demonstrates discrimination within that specific single-gas protocol, but it should not be interpreted as universal intrinsic specificity toward NO_2_. The broader selectivity assessment must distinguish intrinsic chemistry from catalytic filtering, operating-state discrimination, and computational classification and should ultimately be validated in realistic mixtures [31].

Many studies compare equal concentrations of isolated gases, even when realistic interferent concentrations differ by orders of magnitude. Future testing should include mixtures containing NO, O_3_, SO_2_, CO, NH_3_, VOCs, and humidity at application-relevant ratios; sequence-dependent poisoning; and recovery after interferent exposure. For breath or wearable systems, water vapor and CO_2_ should be treated as dominant matrix components rather than optional interferents. Selectivity should be reported as calibration error in mixtures, not just as a bar chart of single-gas response.

Selectivity metrics should also distinguish qualitative discrimination from quantitative accuracy. A receptor may respond more strongly to NO_2_ than to a single interferent yet still produce unacceptable concentration bias when both gases are present. For array-based or algorithm-assisted sensing, blind mixtures and held-out devices are more informative than single-gas bar charts.

### 6.3. Recovery, Hysteresis, Drift, and Overload Tolerance

Fast response does not imply reversible sensing. Strong NO_2_ adsorption can generate a large signal but delay desorption, producing incomplete baseline recovery, hysteresis, and cumulative calibration error. Rapid systems include Ag-WO_3_ [76], MXene/CLFO [51], CuO/rGO [50,110], red-light CuO/MoS_2_ [89], and CdTe/ZnO@porous-Si [66]. Slow or incomplete recovery persists in Rh-ZnO [70], dark In_2_O_3_ nanowires [41], thermally oxidized In_2_O_3_ films [107], SnO_2_/SnS_2_ [65], SnS_2_-rGO [86], porous P3HT@SEBS [127], and hydrangea-like CuO [111].

Thermal, optical, and electrical reset methods should be evaluated as part of the complete sensing cycle. UV-assisted Au-MoS_2_ [81] and In_2_O_3_/MXene [88], visible-light-regenerated In_2_O_3_ [41], pulse-heated MOF-In_2_O_3_ [73], SnO_2_ memristor-heater/CNT [13], and gate-bias-assisted In_2_O_3_ TFT recovery [90] show that adsorption and regeneration can be separated in time. The relevant metric is not only recovery time but recovery fraction, regeneration energy, overshoot, baseline noise after reset, and performance after repeated pulses. Irreversible or cumulative adsorption should be tracked separately through residual baseline offset after a defined purge, cycle-to-cycle signal loss, sensitivity retention, and post-stress recalibration; an electrical return toward baseline does not by itself prove complete molecular desorption.

**Figure 17:**
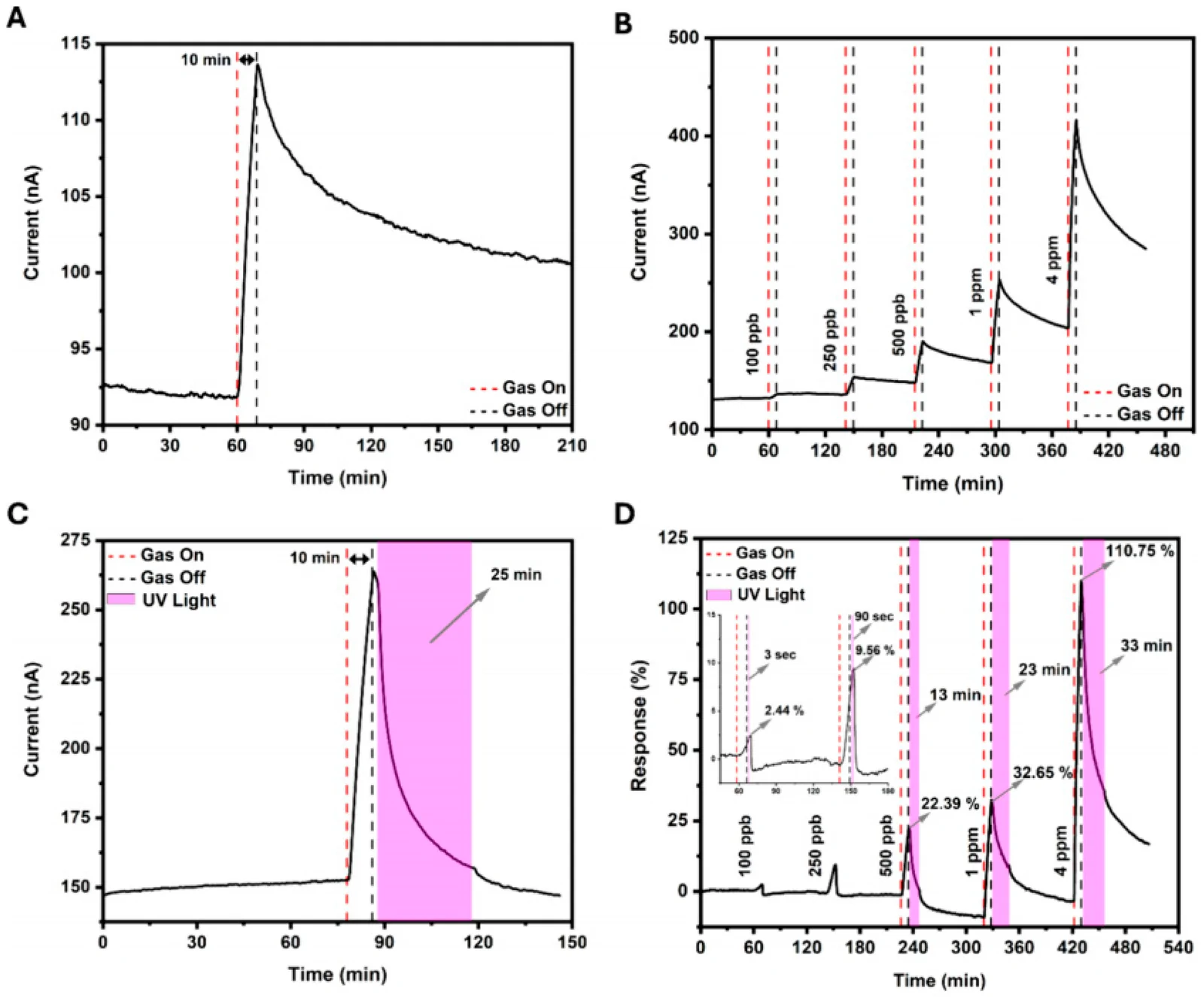
(**A**) Dynamic current characteristic curve of the FePcF_16_–rGO hybrid sensor exposed to 500 ppb NO_2_ (without UV irradiation). (**B**) Dynamic current characteristic curve of the FePcF_16_–rGO hybrid sensor exposed to different concentrations of NO_2_ (0.1, 0.25, 0.5, 1.0, and 4 ppm) without UV irradiation. (**C**) Dynamic current characteristic curve of the FePcF_16_–rGO hybrid sensor exposed to 1 ppm NO_2_ (recovered with UV irradiation). (**D**) Dynamic response characteristic curve of the FePcF_16_–rGO hybrid sensor exposed to different concentrations of NO_2_ (0.1, 0.25, 0.5, 1.0, and 4 ppm) with UV irradiation recovery. All panels adapted with permission from Ref. [32].

Figure 17 directly separates sensing and regeneration in the FePcF_16_–rGO hybrid. Without UV assistance, NO_2_ produces a concentration-dependent current increase characteristic of the p-type hybrid, but the adsorbed gas is sufficiently persistent that the sensor does not recover independently even after prolonged N_2_ purging. When UV light is applied after exposure, photoexcitation increases carrier population and conductivity and promotes desorption, allowing the current to return rapidly toward its baseline. The repeated concentration cycles with UV-assisted recovery therefore demonstrate a useful operating concept: the receptor can remain unilluminated during the analytical exposure and receive a short optical reset only when regeneration is required. This separates measurement from recovery, reducing the risk that continuous illumination changes the adsorption equilibrium or unnecessarily increases energy consumption [32].

Overload tolerance is a distinct requirement. The In_2_O_3_-In_2_(MoO_4_)_3_ shock experiment [104] tests whether a low-limit sensor survives a 10,000 ppm excursion rather than only repeating benign cycles. Future protocols should include a defined overload concentration and duration, time to recover calibration, irreversible sensitivity loss, and post-stress selectivity. Drift should then be decomposed into reversible humidity or temperature dependence, adsorbate accumulation, catalyst or vacancy evolution, contact degradation, and packaging changes.

Recovery should additionally be treated as a concentration-history problem. Low-level exposure and overload can populate different adsorption states, so a single T90 recovery value does not necessarily describe reset after a realistic concentration excursion. This would make slowly recovering systems such as Rh-ZnO, SnS_2_-rGO, and hydrangea-like CuO more directly comparable with visible-light, UV, pulse-heated, or gate-bias-assisted regeneration strategies [41,70,86,90,111].

### 6.4. Gas Delivery, Flow, Chamber Dynamics, and Exposure History

Gas delivery is a measurement variable rather than a neutral background condition. In a direct comparison of ZnO, a tetrazine polymer, and WS_2_, increasing total flow from 100 to 1000 sccm increased the magnitude of every measurable NO_2_ response and shortened most response and recovery times despite different carrier types and structures [16]. For ZnO at 250 °C, the 10 ppm response rose to 1416% at 1000 sccm, while the response time fell to approximately 109 s. The changes reflect chamber exchange, analyte flux, boundary-layer thickness, and competition between adsorption and desorption.

Static injection, sealed-bottle evaporation, dynamic mass-flow delivery, open-air exposure, and breath streams impose different concentration–time profiles. A nominal 1 ppm step may reach the film slowly in a large chamber, overshoot in a small volume, or be diluted by dead volume and leaks. Open-environment monitors experience turbulence and wind speed, while mask or breath sensors experience high humidity, pulsatile flow, and condensation. Therefore, laboratory concentration cannot be interpreted independently of residence time and application-specific transport.

At minimum, studies should report total flow, balance gas, chamber volume and geometry, sensor position, pressure, dead volume, tubing material, injection waveform, exposure/purge duration, and estimated exchange time. Comparative studies should either match transport conditions or normalize them with a validated chamber model. Exposure history should also be recorded because previous high-concentration or interferent pulses can alter adsorption sites and apparent response in subsequent cycles. Software or machine learning compensation cannot substitute for physically defined transport conditions; hardware-level control and characterization of flow, chamber exchange, dead volume, and inlet geometry should precede model-based correction.

Flow sensitivity also complicates the interpretation of response and recovery time because the measured transient may be limited by chamber exchange rather than surface reaction kinetics. The direct flow rate comparison [16] demonstrates that nominally identical NO_2_ concentrations can yield different signal amplitudes and time constants when analyte flux changes. For miniaturized cells, the dead volume and exchange time should be reported together with the mass-flow-controller setpoint; for open-air or wearable platforms, local air speed, sensor orientation, and inlet geometry can be equally important. Without these descriptors, cross-study comparisons can incorrectly attribute transport effects to material chemistry.

### 6.5. Reliability, Aging, Device Variability, and Signal Quality

Long-term stability is not equivalent to repeating a small number of identical cycles. Material aging can involve grain growth, phase conversion, catalyst oxidation or agglomeration, vacancy redistribution, polymer relaxation, and surface contamination. Device aging adds electrode diffusion, contact resistance change, heater cracking, membrane stress, adhesive degradation, and encapsulation leakage. The Ag-ZnO study [17] directly links 60-day signal loss to vacancy evolution, Ag oxidation, increased dark conductivity, and reduced photoconductivity, while the SnO_2_ memristor-heater study [13] shows that heater material and thickness determine cycle-to-cycle thermal variability.

Reliability should be quantified across multiple devices and synthesis batches. Recommended outputs include initial yield, device-to-device coefficient of variation, calibration-slope distribution, baseline drift per day, recovery fraction, failure mode, and retained response after defined thermal, optical, humidity, bending, or overload doses. A single device tested for 30 days cannot establish manufacturing reproducibility, and microscopy of one representative sample cannot substitute for electrical statistics. For repeated adsorption studies, irreversible baseline shift and retained sensitivity after defined cumulative exposure should be reported alongside conventional recovery time.

System-level signal quality requires equally transparent definitions. The Smart Breath Sentinel paper [14] reports an SNR of 574,000 in the abstract but 57,400 in the main text; the review records the inconsistency rather than selecting one value. Circuit-integrated studies should report raw sensor resistance or current, analog front-end gain, sampling bandwidth, filtering, noise window, SNR equation, threshold logic, and latency. Signal processing may improve apparent stability, but raw data are needed to distinguish true receptor performance from amplification, smoothing, or model-based correction.

Reliability is therefore most useful when partitioned into receptor stability, activation-component stability, mechanical/electrical integrity, and calibration stability. Ag-ZnO shows that the photosensitive receptor itself evolves with cumulative illumination, whereas the SnO_2_ memristor-heater/CNT platform isolates heater thickness and thermal cycling as independent reliability variables [13,17]. Flexible In_2_O_3_/MXene and rGO@In_2_O_3_ add water exposure and bending as mechanical/environmental stressors [88,108]. A deployment-oriented aging study should define the applied stress dose and identify which subsystem changes when sensitivity, baseline, recovery, or noise drifts.

### 6.6. Calibration Transfer, Coupled-Environment Validation, and Field Uncertainty

Environmental variables rarely act independently in deployment. Humidity alters adsorption and conduction, flow changes analyte delivery, temperature changes reaction kinetics and baseline resistance, and prior exposure can modify the next cycle. Compensation models trained on one chamber condition can therefore fail when several variables shift simultaneously. The flow rate study, flexible In_2_O_3_/MXene and rGO@In_2_O_3_ platforms, ZITO MOSFET, and Smart Breath Sentinel collectively indicate that transport, humidity, device state, and electronics must be treated as a coupled calibration problem rather than as independent correction factors [12,14,16,88,108].

Calibration transfer should be tested across devices, batches, days, and environmental states. Microscopic differences in grain boundaries, receptor loading, contact resistance, and baseline characteristics can create device-specific domains that are not captured by temperature or RH covariates alone. For a field-oriented sensor, a strong protocol therefore requires device-separated validation, co-location with a traceable reference analyzer, controlled RH/temperature transitions, representative interferent mixtures, and repeated calibration after overload or regeneration. Compensation should be validated on previously unseen devices and conditions, with uncertainty reporting; otherwise, low prediction error may reflect chamber- or device-specific correlations rather than transferable NO_2_ quantification [14,15,51].

The final output should include an uncertainty statement that separates calibration uncertainty, repeatability, environmental sensitivity, drift, flow or sampling uncertainty, and algorithmic uncertainty where applicable. Field readiness is better demonstrated by bounded concentration error over a defined operating envelope than by a single best-case response value. Reporting failure or out-of-range flags is also important: when humidity, temperature, flow, or baseline resistance leaves the validated region, the system should indicate that concentration estimates are no longer reliable. The environmental effects, reliability issues, and deployment-relevant validation requirements discussed in Section 6 are summarized in Table 4.

**Table 4:** Environmental interference, stability, and deployment-relevant validation for NO_2_ sensors.

Evaluation Domain	Main Challenge or Observed Effect	Representative Mitigation/Design Response	Recommended Validation or Reporting	References
Humidity and condensation	Competitive adsorption, hydroxylation, baseline shift, conductive-network doping/swelling, or—in some hybrids—humidity-assisted reactive intermediates.	Hydrophobic/permeable barriers; catalytic filtering; local heating; RH/temperature co-sensing; humidity-tolerant hybrid interfaces.	Dynamic RH ramps; near-condensation exposure; response and baseline recovery after RH steps; calibration error under changing humidity.	[12,13,14,31,82,83,88,108,118,131]
Interferents and gas mixtures	Single-gas selectivity can overestimate performance in mixed atmospheres; selectivity can change with temperature, illumination, bias, or prior exposure.	Catalytic pre-filters; temperature/bias/illumination modulation; chemically selective interfaces; sequential/array readout.	Application-relevant gas ratios; blind mixtures; sequence effects; post-interferent recovery; cross-gas calibration at each operating state.	[31,63,73,74,83,87,89,90,107]
Recovery, hysteresis, and overload	Strong adsorption can yield large response but incomplete reset, hysteresis, and concentration-history dependence.	Thermal, optical, or electrical regeneration; pulse heating; gate-bias reset; recovery-only UV/visible-light operation.	Recovery fraction, residual baseline offset, regeneration energy, overshoot, cycle-to-cycle loss, and defined overload/recalibration protocol.	[30,32,41,70,73,81,86,88,90,104,107,108,111]
Flow, chamber, and transport	Signal magnitude and kinetics can change with total flow, chamber exchange, dead volume, boundary-layer transport, and inlet geometry.	Low-dead-volume cells; controlled dynamic flow; transport-matched comparison; explicit sampling architecture.	Total flow, chamber volume/geometry, exchange time, sensor position, gas waveform, and open-air speed/orientation when applicable.	[16,83,130,131]
Aging and device variability	Receptor chemistry, dopants/catalysts, optical activation, heaters, contacts, flexible substrates, and packaging can drift through different failure mechanisms.	Subsystem-specific durability design; stable activation source; low-drift hybridization; robust heater/substrate selection; batch/process control.	Multi-device and multi-batch statistics; cumulative optical/thermal/mechanical dose; retained response, baseline drift, failure mode, and recalibration.	[13,17,25,26,28,30,32,88,108,118]
Calibration, signal quality, and transfer	Filtering or learned correction can hide raw drift; models may capture chamber/device signatures rather than chemistry.	Co-located environmental channels; transparent front-end characterization; sequential/e-nose features; time-series alignment with external references.	Raw signal and noise definition; device/day-separated validation; external reference co-location; uncertainty, transfer testing, and out-of-range detection.	[14,15,51,87,131]

## 7. Performance Benchmarking, Reporting, and Application-Specific Design

### 7.1. Response Definitions and Cross-Study Comparability

The response conventions used in this literature can differ by orders of magnitude even for the same resistance change. A ratio of 10 expressed as Rg/Ra corresponds to a 900% increase when converted to (R_g_ − R_a_)/R_a_ × 100%, whereas a p-type paper may report the inverse ratio. Conversion is acceptable only when the direction, baseline, and denominator are explicit. The response conventions, associated comparison risks, and recommended treatments are summarized in Table 5.

**Table 5:** Response conventions and recommended treatment.

Reported Form	Typical Context	Comparison Risk	Recommended Treatment	References
R_g_/R_a_	n-type oxidizing-gas response	Looks numerically larger than percent change	Preserve ratio; convert only when explicit Ra and Rg are available.	[20,25,38,43]
R_a_/R_g_	p-type oxidizing-gas response	Opposite response direction from n-type convention	State conduction type and sign of the resistance change.	[27,50,110,111]
|R_g_-R_a_|/R_a_ × 100%	Relative resistance change	Percent and ratio values are often mixed across papers	Keep percentage sign and denominator visible; retain the source-paper definition.	[30,31,32,80,88,116]
Current or threshold-voltage shift	FET/TFT platform	Not directly comparable with resistance ratios	Report gate/drain bias, transconductance, threshold shift, layer count, and readout condition.	[12,29,81,90,103,129]
Photoacoustic amplitude/demodulated relative signal	Optical absorption with acoustic transduction	Sensitivity depends on optical spectrum, background signal, flow, and averaging	Report source spectrum/power, modulation, cell geometry, flow, background correction, acoustic readout, and averaging time.	[3,130]
Flow-conditioned response/kinetics	Dynamic chamber or open-flow chemiresistor	The same nominal concentration can yield different response and T90 at different flows	Report total flow, chamber volume, residence/exchange time, geometry, and gas waveform.	[16]
Voltage output/SNR/alarm threshold	Circuit-integrated or wireless sensor node	Depends on gain, bandwidth, filtering, threshold logic, and SNR definition	Report raw receptor signal, front-end transfer function, noise window, filtering, and threshold logic.	[11,14,15,51]
Time-series corrected concentration	Field-calibrated low-cost sensor	Apparent accuracy can depend on preprocessing, environmental covariates, and train/test leakage	Report raw and corrected series, environmental inputs, split strategy, external reference, uncertainty, and transfer to unseen periods/devices.	[131]
Sequential/temperature-programmed feature vector	MEMS array or electronic-nose operation	Exposure order and thermal history can change surface chemistry and the learned feature space	Report temperature trajectory, exposure sequence, recovery interval, feature extraction, and blind-mixture validation.	[87]

### 7.2. Minimum Reporting Requirements for Reproducible Comparison

The requirements include material composition, quantitative loading, synthesis batch, post-treatment, active mass/area, and film thickness. The substrate, electrode material, finger width/gap, contact method, heater/light geometry, packaging, and electrical bias are also important. For optical or photoacoustic sensors, they are as follows: source wavelength and power, modulation waveform/frequency, absorption path, resonator geometry and Q factor, microphone/detector configuration, flow, and integration time. For photoactivated or pulse-heated sensors, these include the following: cumulative optical/thermal dose, source wavelength and irradiance at the film, pulse duration, heater resistance/temperature calibration, recovery energy, source aging, and pre/post-test dark conductivity. NO_2_ concentration, source gas, dilution gas, total flow, chamber volume and geometry, sensor position, static/dynamic delivery, residence/exchange time, injection waveform, and exposure/purge duration are likewise necessary.

Temperature and RH at the sensing film, stabilization time, and humidity-generation method.

Original response equation, sign of change, sampling rate, filtering, and response/recovery threshold.

Calibration range; lowest experimentally tested concentration; calculated/extrapolated LOD where applicable; noise-equivalent concentration only when supported by measured noise/SNR; replicate cycles; number of devices/batches; signal quality at the range limits; and uncertainty.

Selectivity under realistic concentration ratios and mixtures, including humidity transients and poisoning tests.

Recovery fraction, hysteresis, drift, long-term duration, overload protocol, and post-stress recalibration.

Average system power and energy per measurement cycle, including regeneration, readout, and wireless transmission.

Raw or machine-readable response curves with metadata sufficient for independent normalization.

For circuit-integrated systems, the following are necessary: raw sensor resistance/current, analog front-end gain, sampling bandwidth, filtering, noise definition, SNR equation, threshold logic, humidity/temperature compensation, and wireless packet latency.

For flow-sensitive comparisons, the requirements are as follows: repeat at application-relevant flow rates or demonstrate transport independence; report pressure drop and open-environment air speed when applicable.

For data-driven arrays, a train/validation/test split, device and day separation, preprocessing, model hyperparameters, uncertainty, external validation, and out-of-distribution testing are important.

### 7.3. Application-Specific Design and Validation Rules

Application-oriented design should first define the exposure scenario, required concentration range, time scale, and activation/regeneration budget. Ambient monitoring prioritizes low-ppb accuracy and calibration stability; industrial safety prioritizes rapid alarm and overload recovery; and wearable or personal-exposure platforms add mechanical compatibility, humidity robustness, and low average power. Because room-temperature receptors may still require optical, thermal, or electrical regeneration, energy per complete measurement cycle—not operating temperature alone—is the more relevant metric for battery-powered operation.

Representative platforms illustrate why application guidance must be evidence-based, and why the few directly reported power figures are more informative than temperature-based estimates. The flexible WO_3_/MoS_2_ system [11] adds bending-compatible integration and an alarm/readout function but still operates continuously at 125 °C; the CNT/SnO_2_ memristor-heater platform [13] instead keeps the CNT receptor at room temperature and uses pulse heating only for recovery, shifting the energy burden from continuous sensing to regeneration. As a concrete device-level reference, the localized In_2_O_3_ microheater of Ref. [129] detects 25 ppb while its heater consumes approximately 0.84 mW at 2 V and 50% duty cycle, showing that thermal isolation and pulsed operation can bring true operating power into the sub-milliwatt range. Flexible In_2_O_3_/MXene [88] withstands more than 2000 bending cycles and 70% RH/direct water exposure but requires UV activation, adding an optical-recovery load that offsets its mechanical robustness. Together, these examples show that wearable or battery suitability must be judged from thermal or optical recovery load, humidity robustness, mechanical durability, and system integration considered jointly, rather than from operating temperature alone. Where a source study reports a device-level power or energy-per-cycle value (e.g., ~0.84 mW for Ref. [129]), it is cited directly; because most source papers do not report directly comparable total energy-per-cycle figures—and because such figures depend on heater geometry, thermal mass, duty cycle, and read-out electronics—no cross-platform mW ranking is inferred. The application-oriented design targets and minimum deployment validation requirements are summarized in Table 6.

**Table 6:** Application-oriented design rules and minimum deployment validation.

Application Scenario	Primary Design Target	Promising Strategies/Platform Choices	Minimum Deployment Validation	References
Ambient and distributed monitoring	Trace-concentration accuracy, low drift, humidity tolerance, and low maintenance over long deployments.	Low-temperature oxide heterostructures; porous/vacancy-engineered ZnO/SnO_2_; catalytic filtering; MXene/carbon hybrids; compact photoacoustic sensing; data-driven calibration.	Experimentally demonstrated trace range; dynamic RH; multi-device calibration; long-term drift; co-location with reference analyzer; bounded uncertainty.	[12,25,27,31,39,43,45,49,51,58,70,79,81,83,88,130,131]
Wearable and personal-exposure monitoring	Mechanical compliance, low average power, safe packaging, and reliable response under motion and changing microclimate.	Flexible rGO/oxide and MXene/oxide films; transparent 2D hybrids; CNT/graphene platforms; stretchable polymers; integrated personal-warning nodes.	Bending/strain matrix; perspiration/high-RH exposure; encapsulation/contact stability; average node power; orientation and user-to-user variability.	[11,14,24,30,31,88,108,127,128]
Mask-mounted or breath-adjacent sensing	Fast cycling under high humidity and pulsatile flow with minimal dead volume and clear alarm logic.	Humidity-compensated heated receptor; flexible trace-sensing layer; local environmental sensing and wireless readout.	Breath-like RH/temperature waveform; condensation challenge; pulsatile-flow validation; response latency; false-alarm rate; calibration transfer across users.	[14,31,108,131]
Industrial leak and exhaust monitoring	Fast alarm, wide dynamic range, poisoning resistance, thermal robustness, and recovery after concentration shock.	Heated MOS; vacancy/facet and catalyst engineering; p-n heterojunctions; catalytic filtering; temperature-selective interfaces.	Defined overload concentration/duration; mixed-gas interference; temperature correction; post-shock recalibration; poisoning and long-duration operation.	[25,26,39,42,44,63,70,74,76,83,104,111]
Battery-powered sensor nodes	Low energy per complete sensing cycle while maintaining complete recovery and stable baseline.	Localized microheaters; floating-gate/FET readout; duty-cycled optical reset; CVD graphene or 2D FETs; compact LED/photoacoustic architectures.	Energy per cycle including regeneration/readout/wireless; warm-up and reset latency; heater/light-source endurance; baseline recovery and sleep-mode drift.	[12,13,29,30,32,41,81,88,90,103,129,130]
NO_x_ speciation and multi-gas discrimination	Discriminate NO_2_ from chemically related or coexisting species rather than merely detect an oxidizing gas.	Dual-mode or two-channel photoacoustic detection; catalytic filtering; temperature-dependent selectivity; sequential MEMS operation; sensor arrays.	Crosstalk matrix; realistic-ratio mixtures; state-specific calibration; exposure-order control; optical averaging and background stability.	[3,31,63,73,74,83,87,107,130]
Concentration prediction and electronic-nose operation	Quantitative inference from arrays or partial transients while resisting device/day domain shift.	Composite sensor arrays; temperature-programmed SnO_2_ MEMS; partial-transient modeling; environmental covariates and time-series calibration.	Device/day-separated train–test split; blind mixtures; external test set; uncertainty/out-of-distribution detection; raw-signal retention.	[15,51,87,131]
Dynamic-flow and open-environment sensing	Maintain calibration when analyte delivery, turbulence, or residence time differs from laboratory step exposure.	Low-dead-volume cells; flow-characterized platforms; catalytic-filter front ends; field calibration with environmental metadata.	Application-rate flow sweep; chamber exchange/residence time; air-speed/orientation effects; pressure/RH/temperature transients; waveform reproducibility.	[13,14,16,83,130,131]
Long-term photoactivated or pulse-assisted operation	Preserve sensitivity and recovery while the activation source, catalyst state, heater, or receptor chemistry ages.	Visible/UV activation with controlled dose; recovery-only optical pulses; low-drift organic hybrids; durable heaters; long-term optoelectronic monitoring.	Cumulative photon/thermal dose; dark versus activated baseline; source/heater calibration; surface-state change; multi-device aging and failure-mode reporting.	[13,17,26,28,30,32,85,116]

These design rules are intended as application-specific validation pathways rather than material rankings. Comparison is meaningful only within a defined concentration, humidity, temperature, flow, power, mechanical, and regeneration envelope. For practical deployment, the complete measurement cycle should include stabilization or warm-up, analyte exposure, readout, regeneration, communication, and periodic recalibration. A platform that delivers a moderate response with reproducible calibration, complete recovery, bounded uncertainty, and low life-cycle energy can be more useful than a record-response material tested under a narrow laboratory condition.

Accordingly, application tables should be updated whenever the literature corpus expands: new materials should not be added solely because they report a larger response or an isolated lower concentration but because their demonstrated operating envelope improves one or more deployment requirements such as humidity robustness, recovery completeness, overload tolerance, mechanical durability, flow independence, calibration transfer, or system-level power.

## 8. Research Gaps and Translation Priorities

### 8.1. Mechanism Verification and Standardized Benchmarking

Mechanistic interpretation remains one of the largest sources of uncertainty in NO_2_ sensor research. Changes in resistance or current are often attributed to oxygen vacancies, catalytic spillover, heterojunction barriers, surface depletion, or charge trapping, yet several of these effects can occur simultaneously. Ex situ XPS, microscopy, work-function measurements, and electrical trends are valuable, but they rarely identify the adsorbed nitrogen species or reveal which elementary step limits adsorption, transduction, or desorption. Future studies should combine operando or in situ infrared/Raman spectroscopy, X-ray absorption, Kelvin probe measurements, temperature-programmed or mass-spectrometric desorption, and synchronized electrical readout under the same RH, temperature, illumination, and bias used for sensing [1,20].

Mechanistic claims should also distinguish receptor chemistry from transducer amplification. A larger signal may arise from more adsorption sites, a stronger interfacial barrier modulation, a higher transconductance, or a lower-noise readout even when the number of adsorbed NO_2_ molecules is similar. This distinction is particularly important for heterostructures, FET/MOSFET platforms, catalytic composites, and optically activated devices, where interface density and electrical operating point can dominate the measured response [12,20,76,90,103].

Beyond the reporting rules summarized in Section 7, the field now needs interlaboratory validation. Round-robin studies using common reference films, shared gas-delivery protocols, or reference sensor packages would help separate genuine material effects from chamber, flow, electronics, and analysis artifacts. Such studies would also provide a stronger basis for transferable calibration and uncertainty estimates across laboratories.

### 8.2. Adaptive Regeneration, Life-Cycle Energy, and Actuator Durability

The next generation of low-power NO_2_ sensors is likely to separate the adsorption and regeneration phases rather than maintain continuous thermal or optical activation. Rough-layer ZnO [40], localized microheaters [129], WO_3_ charge-storage FETs [103], In_2_O_3_ TFT pulsed-bias recovery [90], visible-light In_2_O_3_ [41], pulse-heated MOF-In_2_O_3_ [73], red-light CuO/MoS_2_ [89], UV In_2_O_3_/MXene [88], UV-reset Au-MoS_2_ FETs [81], SnO_2_ memristor-heater/CNT [13], UV-reset SnO_x_/SnS [101], APTES@WO_3_-x [85], and UV ZnO/NiO [9] collectively show that regeneration can be treated as an actively controlled part of the measurement cycle.

Future work should therefore report energy per complete measurement cycle rather than only heater power, operating temperature, or optical irradiance. The relevant budget includes warm-up, baseline stabilization, analyte exposure, readout, regeneration, computation, communication, and any post-reset settling period. A room-temperature receptor that requires long or high-power recovery pulses may consume more life-cycle energy than a mildly heated sensor with rapid and reproducible desorption. Duty-cycle optimization should be evaluated against detection latency, baseline reproducibility, and recovery completeness rather than energy alone.

Adaptive regeneration also creates a control problem. Reset pulses should ideally be triggered by measurable indicators such as incomplete recovery, baseline offset, signal saturation, or accumulated exposure dose, rather than applied at a fixed interval. Durability tests should track cumulative photon or thermal dose, heater resistance and temperature calibration, activation-source aging, catalyst or surface-state evolution, dark conductivity, recovery fraction, and sensitivity after thousands of cycles [13,17,41,116]. Electrical return to the initial baseline should not automatically be interpreted as complete molecular desorption because transient photocarriers or gate-induced charge redistribution can temporarily mask residual adsorbates.

### 8.3. Environmental Robustness, Calibration Transfer, and Field Uncertainty

Humidity, temperature, flow, gas mixtures, and exposure history act jointly in deployment. Water alters adsorption and surface hydroxylation, while flow changes analyte flux and residence time; temperature and prior exposures simultaneously change the kinetics and baseline state. Calibration obtained at one fixed RH and flow condition may therefore fail in open-air, wearable, exhaust, or breath-adjacent environments even when each variable appears manageable in isolation.

Field translation requires more than demonstrating stable cycles in a laboratory chamber. Sensors should be co-located with traceable reference analyzers, challenged by realistic concentration ramps and interferent mixtures, and evaluated across days, devices, seasons, and installation orientations. Calibration-transfer methods should report prediction uncertainty and identify conditions outside the trained operating envelope. Promising integrated approaches include flexible rGO@In_2_O_3_ [108], heater-integrated CNT sensing [13], long-term GaN/graphene operation [116], and the humidity-compensated Smart Breath Sentinel [14], but their broader transferability depends on turbulence, condensation, packaging diffusion, drift, and user- or site-specific variability.

A useful qualification protocol would define an application-specific environmental envelope before sensor optimization begins. For ambient monitoring, this may emphasize low-ppb accuracy, daily RH/temperature cycles, ozone or VOC interference, and long drift intervals; occupational or exhaust sensing should emphasize overload recovery, temperature variation, poisoning, and rapid alarm latency; wearable or mask-mounted systems require mechanical motion, high local humidity, condensation, and orientation-dependent flow. Reporting uncertainty across this full envelope is more informative than quoting a single best response measured under nominal conditions.

### 8.4. Reproducible Manufacturing, Scale-Up, and Sustainability

High-performance composites often depend on narrow loading windows, multistep synthesis, and carefully tuned post-treatment, so laboratory optimization must be separated from manufacturability. Future reports should quantify process yield, batch variation, active-mass distribution, film thickness, adhesion, electrode contact resistance, precursor utilization, solvent or etchant hazards, annealing energy, and device-to-device variability. Scalable routes represented in the reviewed literature include thermal oxidation of evaporated In films [107], solvothermal/nitridation GaN fibers [75], controlled partial oxidation [65,101], MOF-derived processing [9,73,118], CuO/rGO solution synthesis [110,117], flexible In_2_O_3_/MXene deposition [88], breath-figure polymer films [127], microwave Au functionalization [81], co-sputtered ZITO [12], solution-grown hydrangea CuO [111], and spin-coated SnO_2_ [38].

Manufacturing claims should be linked to statistical electrical performance rather than inferred from representative microscopy. Process-control parameters such as deposition thickness, catalyst loading, porosity, annealing profile, interface composition, and contact geometry should be connected to calibration-slope distributions and failure rates across multiple devices and batches. This would reveal whether a reported optimum is robust to normal fabrication variation or depends on a narrow, difficult-to-control process window.

Sustainability should also enter platform selection earlier. Life-cycle assessment should consider scarce or toxic elements, strong-acid or fluoride processing, noble-metal loading, high-temperature calcination, disposable substrates, replaceable light or heater components, calibration labor, and end-of-life handling. A sensor with a slightly smaller response may be more deployable if it offers uniform deposition, lower life-cycle energy, safer chemistry, stable calibration, repairable electronics, and simpler packaging. These criteria become increasingly important as NO_2_ sensing moves from single laboratory devices toward dense distributed networks and wearable systems.

### 8.5. Stage-Gated Translation from Material Screening to Qualified Sensor Systems

A practical translation pathway should be stage-gated. Material screening should first establish reproducible adsorption/transduction behavior and a realistic concentration range. Device-level development should then optimize electrode geometry, bias, heater or illumination control, packaging, and recovery. Environmental qualification should follow using dynamic RH, temperature, interferents, flow, overload, and aging. Only after these stages should field co-location, calibration transfer, wireless operation, maintenance interval, and total system energy be used to judge deployment readiness. This sequence reduces the risk of optimizing sophisticated electronics around a receptor whose environmental behavior is still poorly constrained.

Progress through these stages should be judged by multi-objective performance rather than a single material metric: experimentally demonstrated concentration range, reversible kinetics, selectivity, drift, environmental robustness, life-cycle energy, fabrication yield, mechanical durability, calibration transfer, and cost. Shared raw signals and environmental metadata would further support cross-laboratory normalization, external machine learning validation, and meaningful comparison of complete sensing cycles.

## 9. Conclusions

This review shows that progress in NO_2_ sensing is no longer governed by the receptor material alone. High-performing platforms increasingly combine surface adsorption chemistry with controlled defects, heterojunction or Schottky barriers, conductive networks, catalytic sites, and device-level amplification. ZnO, SnO_2_, WO_3_, and In_2_O_3_ remain central metal-oxide platforms because their morphology, vacancy population, interface chemistry, and processing routes can be widely tuned, while CuO, Co_3_O_4_, GaN/Ga_2_O_3_, carbon materials, TMDs, MXenes, MOF-derived structures, and organic semiconductors broaden the accessible operating temperature, mechanical format, and transduction mechanism [1,5,6,18].

A second conclusion is that low-temperature or room-temperature operation should be evaluated over the complete sensing cycle. Optical activation, localized heating, floating-gate control, pulsed bias, and memristor heaters can reduce steady-state power or accelerate recovery, but they also introduce activation energy, control complexity, source aging, and additional failure modes [9,12,13,17,41,81,88,90,103,129]. The most meaningful energy metric is therefore energy per validated measurement cycle, including stabilization, exposure, readout, regeneration, computation, and communication.

The literature also makes clear that record response and isolated trace-concentration claims are insufficient indicators of deployability. The experimentally generated concentration range should be interpreted together with response/recovery time, recovery fraction, humidity dependence, gas-flow conditions, drift, device-to-device variation, overload tolerance, and raw signal quality [14,16,49,70,101,104]. Dynamic humidity and transport effects can alter both magnitude and kinetics, while incomplete desorption can create history-dependent calibration even when the initial response is large.

Accordingly, the preferred sensing architecture depends on the application envelope. Ambient monitoring prioritizes trace-level accuracy, calibration stability, and long-term humidity robustness; industrial and exhaust monitoring require wide dynamic range, poisoning resistance, and recovery after concentration shocks; battery-powered nodes require low life-cycle energy and durable regeneration; wearable and mask-mounted systems add bending, condensation, pulsatile flow, packaging, and user variability; and multi-gas or NO_x_ applications may justify sensor arrays or spectroscopic transducers [3,11,14,15,51,104,108]. A universal material ranking is therefore less useful than application-specific qualification against a defined set of environmental and system constraints.

The central message of this review is that the transition from a high-response material to a trusted NO_2_ sensor system requires a shift from isolated sensitivity metrics to application-specific validation of the complete measurement cycle. Receptor chemistry and interfaces determine how NO_2_ perturbs charge; the transducer determines how that perturbation is read; environmental and gas-delivery conditions determine what reaches the receptor; regeneration and aging determine repeatability; and calibration, uncertainty, and system integration determine whether the output can support a real measurement decision. Advances will be most consequential when these layers are optimized and reported together, enabling reproducible, energy-efficient, field-validated NO_2_ monitoring.

## Figures and Tables

**Table 2:** Summary of functional-material platforms for NO_2_ sensing.

Material Platform	Representative Engineering Routes	Main Sensing Strengths	Persistent Limitations/Trade-Offs	References
ZnO	Porous/hierarchical morphology; vacancy/facet control; Rh/Ce doping; Au catalysis; rGO/MXene/g-C_3_N_4_ coupling; CuO-ZnO heterojunctions; ZnO catalytic filters	Broad synthesis tunability, strong surface/defect control, and trace-concentration response in optimized porous and heterointerface structures	Moderate heating is still common; recovery, humidity sensitivity, catalyst/defect aging, pore reproducibility, and interface uniformity remain critical.	[17,19,34,36,37,39,40,42,55,60,66,70,71,78,83,92,93,94,95]
SnO_2_	Pd/rGO functionalization; oxide and oxide–sulfide heterointerfaces; MXene/CNT coupling; porous/faceted nanocrystals; Dy doping; MEMS/ALD processing; catalytic-filter integration	Chemically stable and scalable; supports low-temperature and room-temperature sensing when interfaces, defects, and conductive pathways are engineered	Slow or incomplete recovery can persist; aggregation, gas trapping, cross-sensitivity, thermal state, and flow-dependent kinetics complicate comparison.	[25,26,33,38,43,47,52,58,62,65,83,86,87,96,97,98,99,100,102]
WO_3_	Y/Ag/Co defect engineering; APTES functionalization; MoS_2_ or WS_2_ coupling; floating-gate FET control	Strong affinity to oxidizing gases and high barrier sensitivity after defect, catalyst, or heterojunction engineering	Often requires heating or illumination; strong adsorption can penalize recovery, while functionalization and heterostructure processing add stability concerns.	[11,44,68,72,76,85,103]
In_2_O_3_	Mixed phases; QD sensitization; sulfide/oxide and GaN interfaces; MXene/rGO coupling; visible/UV regeneration; TFT integration; vertically stacked In_2_O_3_/In_2_S_3_	High response at low temperature and strong compatibility with porous, flexible, optical, and transistor platforms	Humidity dependence, dark recovery, baseline resistance, activation energy, and reproducibility of ultrathin interfaces must be controlled.	[15,41,45,63,64,69,73,77,88,104,105,106,107,108,109]
Other oxides and wide-bandgap semiconductors	CuO, NiO, CeO_2_, Co_3_O_4_, GaN, and Ga_2_O_3_; p-n heterojunctions, noble metals, porous structures, and MEMS integration	Complementary p-type response, catalyst compatibility, and robust operation in thermally or chemically demanding environments	Operating-temperature requirements and recovery vary widely; p-type hybrid response can be strongly altered by humidity and conductive-carbon coupling.	[10,27,42,50,59,60,61,74,75,79,80,89,110,111,112,113,114,115,116]
Carbon materials	rGO, CNTs, CVD graphene, laser-induced graphene, N-rGO/NiO, InSe-graphene, and phthalocyanine-rGO hybrids	Room-temperature conductivity, mechanical flexibility, facile patterning, and efficient interfacial charge transport	Humidity response, gas trapping, contact/junction variability, and incomplete recovery require controlled network density and regeneration.	[13,18,24,27,30,31,32,34,35,47,50,52,53,54,92,102,117,118]
TMDs, MXenes, and mixed-dimensional hybrids	MoS_2_/SnS_2_/GaSe sensing layers; Ti_3_C_2_Tx interfaces; oxide-TMD heterojunctions; Au decoration; WS_2_/oxide hybrids; PTCDI/MXene; MoS_2_ FETs	High surface exposure, tunable band alignment, flexible integration, and effective coupling to optical or gate modulation	Pristine 2D materials may recover slowly or degrade; contact resistance, oxidation, loading, photoactivation, and layer count require control.	[7,11,23,29,31,43,44,45,46,48,51,55,56,57,58,62,81,82,84,88,119,120,121,122,123,124,125,126]
Porous, MOF-derived, and biomimetic structures	MOF pyrolysis, engineered open porosity, hierarchical rough films, self-supporting growth, natural/sacrificial templates, and catalytic-filter nanotube arrays	Large accessible area, interconnected diffusion pathways, and convenient creation of mixed-metal or heterojunction architectures	Pore preservation, calcination energy, precursor/solvent use, tortuosity, batch reproducibility, and filter/receptor crosstalk matter for scale-up.	[9,36,37,39,40,49,51,80,83,111,118]
Conducting polymers and stretchable organics	PANI/BP composites, nanoporous P3HT@SEBS, molecular–organic/MXene hybrids, and FePcF_16_–rGO receptors	Intrinsic low-temperature operation, tunable molecular adsorption, and mechanical compliance for flexible sensing	Humidity-dependent doping/swelling, baseline drift, batch-dependent morphology, and incomplete recovery remain major barriers.	[1,28,32,46,127]

**Table 3:** Summary of device architectures and system-level sensing strategies.

Architecture/Strategy	Representative Implementation	Primary Advantage	Key Validation Requirement	**References**
Interdigitated electrodes, flexible substrates, and direct writing	Printed/sprayed oxide–carbon films, LIG on polyimide, flexible WO_3_/MoS_2_, In_2_O_3_/MXene, rGO@In_2_O_3_, stretchable polymers, CVD graphene	Simple fabrication, mechanical compliance, transparent/wearable integration, and compatibility with scalable deposition	Report bending radius/strain, cycle count, contact stability, encapsulation, transmittance when relevant, and sensing before/after mechanical stress.	[11,14,24,30,34,66,77,88,96,97,102,108,127,128]
MEMS and localized heating	CuO/MEMS, Au/Co_3_O_4_ MEMS, localized In_2_O_3_ microheater FET, SnO_2_ memristor-heater/CNT, ALD-SnO_2_ MEMS e-nose arrays	Small thermal mass enables low average power, rapid temperature control, and programmed temperature fingerprints or duty-cycled regeneration	Benchmark heater calibration, thermal uniformity, energy per pulse/cycle, programmed temperature trajectory, recovery fraction, and heater-cycle durability.	[13,79,87,112,129]
FET/TFT/MOSFET and floating-gate control	WO_3_ floating-gate FET, In_2_O_3_ TFT with pulsed-bias recovery, Au-MoS_2_ FET, co-sputtered ZITO MOSFET, back-gated MoS_2_ FET	Gate bias and transconductance provide electrical amplification plus an additional variable for sensitivity, selectivity, or recovery	Report bias state, threshold shift, transconductance, gate history, microheater state, layer count, and recovery protocol; do not compare directly with resistance ratios.	[12,29,81,90,103]
Optical and hybrid regeneration	UV/visible/red-light activation, recovery-only light pulses, oxide-TMD photoactivation, CVD-graphene UV conditioning, FePcF_16_–rGO UV reset, micro-LED-compatible concepts	Allows lower-temperature sensing and can decouple adsorption from regeneration when duty-cycled	Report wavelength, irradiance, duty cycle, photoconductive baseline, recovery fraction, source aging, and cumulative photon dose.	[7,9,17,30,32,40,41,52,85,88,89,91,116]
Photoacoustic/spectroscopic transduction	Dual-resonant NO/NO_2_ photoacoustic cell and two-channel LED/cantilever-enhanced photoacoustic sensing	Provides spectrally selective NO_2_ or NO_x_ measurement without relying on surface-resistance modulation	Report optical power/spectrum, modulation, flow, cell geometry, background subtraction, acoustic readout, and averaging time.	[3,130]
AI-enabled IoT, e-nose, and distributed sensing	Wireless/wearable nodes, humidity-compensated mask system, SnO_2_ temperature-programmed MEMS arrays, partial-transient deep learning, ANN-based field calibration	Combines sensing, compensation, discrimination, concentration inference, alarms, and wireless deployment at system level	Separate receptor, front-end, algorithm, and communication performance; use device/day-separated validation, external references, uncertainty, and out-of-distribution testing.	[1,7,11,14,15,16,51,87,96,131]
Catalytic pre-filters and chemically selective front ends	ZnO-nanosheet filter over Pd/SnO_2_ nanotube array with top–bottom electrode isolation	Improves chemical specificity before electrical transduction and can reduce humidity/interferent impact	Validate filter conversion efficiency, pressure/transport penalty, thermal budget, receptor/filter crosstalk, and mixed-gas performance over time.	[83]

## Data Availability

No new data were generated in this review. Data sharing is not applicable to this article.
